# New Insight into Quinoa Seed Quality under Salinity: Changes in Proteomic and Amino Acid Profiles, Phenolic Content, and Antioxidant Activity of Protein Extracts

**DOI:** 10.3389/fpls.2016.00656

**Published:** 2016-05-18

**Authors:** Iris Aloisi, Luigi Parrotta, Karina B. Ruiz, Claudia Landi, Luca Bini, Giampiero Cai, Stefania Biondi, Stefano Del Duca

**Affiliations:** ^1^Department of Biological, Geological and Environmental Sciences, University of BolognaBologna, Italy; ^2^Departamento de Producción Agrícola, Universidad de ChileSantiago, Chile; ^3^Department of Life Sciences, University of SienaSiena, Italy

**Keywords:** antioxidant activity, *Chenopodium quinoa*, polyphenols, salt stress, seed storage proteins

## Abstract

Quinoa (*Chenopodium quinoa* Willd) is an ancient Andean seed-producing crop well known for its exceptional nutritional properties and resistance to adverse environmental conditions, such as salinity and drought. Seed storage proteins, amino acid composition, and bioactive compounds play a crucial role in determining the nutritional value of quinoa. Seeds harvested from three Chilean landraces of quinoa, one belonging to the *salares* ecotype (R49) and two to the coastal-lowlands ecotype, VI-1 and Villarrica (VR), exposed to two levels of salinity (100 and 300 mM NaCl) were used to conduct a sequential extraction of storage proteins in order to obtain fractions enriched in albumins/globulins, 11S globulin and in prolamin-like proteins. The composition of the resulting protein fractions was analyzed by one- and two-dimensional polyacrylamide gel electrophoresis. Results confirmed a high polymorphism in seed storage proteins; the two most representative genotype-specific bands of the albumin/globulin fraction were the 30- and 32-kDa bands, while the 11S globulin showed genotype-specific polymorphism for the 40- and 42-kDa bands. Spot analysis by mass spectrometry followed by *in silico* analyses were conducted to identify the proteins whose expression changed most significantly in response to salinity in VR. Proteins belonging to several functional categories (i.e., stress protein, metabolism, and storage) were affected by salinity. Other nutritional and functional properties, namely amino acid profiles, total polyphenol (TPC) and flavonoid (TFC) contents, and antioxidant activity (AA) of protein extracts were also analyzed. With the exception of Ala and Met in R49, all amino acids derived from protein hydrolysis were diminished in seeds from salt-treated plants, especially in landrace VI-1. By contrast, several free amino acids were unchanged or increased by salinity in R49 as compared with VR and VI-1, suggesting a greater tolerance in the *salares* landrace. VR had the highest TPC and AA under non-saline conditions. Salinity increased TPC in all three landraces, with the strongest increase occurring in R49, and enhanced radical scavenging capacity in R49 and VR. Overall, results show that salinity deeply altered the seed proteome and amino acid profiles and, in general, increased the concentration of bioactive molecules and AA of protein extracts in a genotype-dependent manner.

## Introduction

Quinoa (*Chenopodium quinoa* Willd., Amaranthaceae) is an Andean seed-producing crop cultivated since ca. 7000 years around Lake Titicaca in the Andean highlands (*altiplano*) from where it spread as far north as Ecuador and down to southern Chile, and from 3800 m above sea level to coastal and lowlands areas. This diversification in terms of native habitats and the wide genetic diversity has led to the identification of five ecotypes: *salares* (salt flats), highlands, inter-Andean valleys, *yungas*, and coastal-lowlands. The *salares* of the Andes are found principally in southern Bolivia, northern Chile, and Argentina. These highland deserts are extremely arid; temperatures often fall well below freezing and quinoa is the only crop that can grow under these edapho-climatic conditions (Fuentes et al., 2009). Other landraces (local varieties) are adapted to totally different environments. For example, in central and southern Chile, quinoa can grow at sea level; here annual rainfall, distributed throughout the year, ranges from 400 to 1500–2000 mm and soils have a high water retention capacity. Thus, quinoa has attracted the attention of scientists since over a decade mainly for its extreme tolerance and adaptability to unfavorable environmental conditions, such as salinity, drought, and frost (Jacobsen et al., 2003). However, different accessions, landraces, and cultivars of quinoa have been shown to exhibit different degrees of tolerance to abiotic, in particular salt stress (Gómez-Pando et al., 2010; Adolf et al., 2012; Peterson and Murphy, 2015).

Consumption of seeds is the most common use of quinoa, which is, therefore, considered a “cereal-like” crop. European and North American consumers are increasingly aware of the exceptional nutritional qualities of quinoa seeds that, together with sprouts, are now considered “functional foods” (Vega-Gálvez et al., 2010). This is because seeds have high protein content and contain all the essential amino acids (Abugoch et al., 2008). In quinoa seeds, protein and lipid reserves are accumulated in embryonic protein and lipid bodies, respectively, which occupy most of the cell (Prego et al., 1998). The classification of seed storage proteins dates from the turn of the century, when Osborne (1924) classified them into groups on the basis of their extraction and solubility, convention still used nowadays for storage proteins from plant seeds in general. These proteins are soluble in water (albumins), dilute saline solution (globulins), alcohol mixtures (prolamins), and dilute acid or alkali (glutelins). The major seed storage proteins include albumins, globulins, and prolamins (Shewry et al., 1995). The main protein fractions in quinoa seeds are globulins and albumins. Brinegar and Goundan (1993) characterized the 11S storage protein, chenopodin, and showed that it is composed of two subunit groups bridged by a disulfide bond, the acidic A subunit group (32–39 kDa) and the basic B subunit group (22–23 kDa). Moreover, they reported that the 11S and 2S (8–9 kDa) polypeptides represented ca. 35 and 37%, respectively of total proteins (Brinegar and Goundan, 1993; Brinegar et al., 1996). The genomic and amino acid sequences of the 11S globulin of quinoa have been reported (Balzotti et al., 2008).

Quinoa seeds are also rich in bioactive compounds, such as vitamins (vitamin B2, vitamin E), carotene, tocopherols, and other molecules exerting antioxidant properties (e.g., phenolics) that scavenge harmful radicals (Paśko et al., 2009; Hirose et al., 2010; Repo-Carrasco-Valencia et al., 2010; Miranda et al., 2014). Diversity in these nutritional traits across genotypes has been reported (González et al., 2011; Vidueiros et al., 2015). In seeds of six quinoa genotypes from three geographical areas of Chile, significant differences in all the parameters analyzed were reported, with VR showing the highest content of protein, and vitamins E and C (Miranda et al., 2013, 2014). Seeds were also described as good sources of antioxidant compounds, although phenolic content, antioxidant and antimicrobial activities also varied among genotypes.

While many studies have been performed to investigate the tolerance of different quinoa genotypes to abiotic stress (mainly salinity) in terms of agronomic performance (growth, yield) and morpho/physiological mechanisms underlying salt tolerance (Adolf et al., 2013; Ruiz et al., 2016b), fewer have been devoted to the effects of high salinity on the nutritional quality of quinoa seeds. Increases or no effects in total protein content have been reported in most cases (Karyotis et al., 2003; Hariadi et al., 2011; Pulvento et al., 2012; Miranda et al., 2013). The effects of drought and salinity on seed phenolic content were investigated and only limited changes in these compounds under reduced irrigation (with or without salinity) were found (Gómez-Caravaca et al., 2012). Recently, increased total polyphenolics content (TPC) and antioxidant activity (AA) in methanol extracts of quinoa seeds harvested from plants grown under salinity has been observed, suggesting that stressful conditions may positively affect the seed's content of these important bioactive compounds (Ruiz et al., 2016a). Flour and the protein concentrate (PC) of seeds of amaranth, a close relative of quinoa, have been shown to contain polyphenols and to possess AA (Escudero et al., 2011). To date, these parameters have not been investigated in quinoa protein extracts.

The purpose of the present work was to investigate changes in the amino acid and protein profiles of seeds obtained from quinoa plants grown under saline conditions. In general, the capacity to accumulate more storage proteins in seeds plays an important role in the initial stages of the next generation, especially germination (Koyro and Eisa, 2008). Our purpose, however, was to conduct a more detailed investigation on the changes occurring under salinity in the relative amounts of different protein fractions (albumin/globulin fraction) and, more specifically, in spot patterns of individual proteins. The proteomic analysis reveals which proteins are involved in the salt-stress response in quinoa, thus contributing to a better understanding of the complex metabolic network involved in stress responses of halophytes (Koyro et al., 2013). In addition, the TPC and AA of protein extracts were evaluated in control and salinized seeds. These features strongly contribute to the nutraceutical properties of quinoa (Repo-Carrasco-Valencia et al., 2010; Abderrahim et al., 2015; Tang et al., 2015), may influence seed longevity (Sano et al., 2015), and can ultimately lead to the production of PCs to be used in the food industry and as dietary complements for their high protein level, functional properties, and low content of anti-nutritional factors (Cordero-De-Los-Santos et al., 2005; Escudero et al., 2011; Castel et al., 2014). To date, the phenolics content and AA of quinoa seed protein extracts have not yet been examined. Given the strong genotypic differences reported for all aspects of quinoa's responses to saline conditions (Adolf et al., 2012; Ruiz et al., 2016b), including nutritional aspects (Miranda et al., 2014; Abderrahim et al., 2015; Tang et al., 2015; Vidueiros et al., 2015), these parameters were comparatively analyzed in different genotypes. Thus, three Chilean landraces originating from contrasting habitats, namely R49, belonging to the *salares* ecotype, and two landraces belonging to the coastal-lowlands ecotype but from different latitudes and altitudes (VI-1, Villarrica), were examined. Seeds were harvested from plants grown in a pot experiment under saline (100 or 300 mM NaCl) and non-saline (0 mM NaCl) conditions.

## Materials and methods

### Plant material

Seeds of three Chilean landraces of *C. quinoa* (Willd.), one belonging to the *salares* ecotype (R49) and two, VI-1 and Villarrica (VR), to the coastal-lowlands ecotype were collected along an altitudinal gradient from the arid northern highland with saline soils (R49, 3800 m a.s.l.) to sea level, and along a latitudinal gradient of ca. 2500 km, from ca. 34°S (VI-1) down to the rainier southern region (ca. 39°S, VR) with higher precipitation and non-saline soils (Peterson and Murphy, 2015). All seeds were obtained from the National Seed Bank of Chile managed by INIA-Intihuasi (Vicuña, Chile).

Vernalized seeds were sown in 20-L plastic pots containing a garden soil:sand (1:1) mixture. When plants had four to six well-expanded leaves (ca. 34 days after sowing) salt treatment was started by irrigating pots weekly with 0, 100, or 300 mM NaCl solutions; all the pots (control and salt-treated) were also watered weekly with 100–200 mL water supplemented with Phostrogen (N:P:K 10:10:27; 0.4 g L^−1^; Bayer Garden, Cambridge, UK). Plants were grown (October to April) in a greenhouse under natural daylight conditions; the temperature was maintained at 23 ± 3°C. Seeds were collected at maturity starting from 91 days and up to 140 days after the first salt treatment, depending on the landrace, weighed, and stored in an air-tight container at 4°C until use.

### Chemicals

All chemicals were obtained from Sigma-Aldrich (Milan, Italy) unless otherwise indicated.

### Seed flour preparation

In order to remove saponins, quinoa seeds were washed repeatedly with cold water until there was no more foam in the wash water, and then dried at 50°C up to 15 ± 3% moisture. The dried seeds were ground to a fine powder using a mortar and pestle. The resulting flour was defatted with hexane under continuous stirring overnight and then air-dried at room temperature. Flours were stored at 4°C until use.

### Preparation of protein fractions

Protein fractions were prepared by a solvent-based sequential extraction following the methods of Ju et al. (2001), Bergamo et al. (2011), Džunková et al. (2011), and Zevallos et al. (2012), with slight modifications. Briefly, the albumin/globulin fraction was obtained by suspending 50 mg of flour in 300 μl of 5% NaCl; the suspension was then homogenized for 5 min and centrifuged at 5500 g for 10 min. The procedure was repeated twice and the supernatants were collected. The flour was further extracted for prolamin-like proteins following the same procedure but using 250 μl of 60% (v/v) aqueous ethanol. The crude acid-soluble globulins and the 11S-enriched fraction were simultaneously isolated following Thanh et al. (1975). Briefly, defatted quinoa flour was extracted with 63 mM Tris-HCl buffer containing 10 mM β-mercaptoethanol, pH 7.8, for 1 h and then centrifuged for 15 min at 9500 × g. The extraction buffer was adjusted to pH 6.6, dialyzed against 63 mM Tris-HCl containing 10 mM β- mercaptoethanol, pH 6.6, at 4°C for at least 4 h and centrifuged for 20 min at 9500 × g. The precipitate corresponded to the crude 11S fraction while the supernatant, which corresponded to the crude acid-soluble globulin fraction, was further purified by adjusting the pH to 4.8. After centrifugation, the precipitate was dispersed in water and the pH was raised to 7.0. For assaying radical scavenging activity, extraction of the 11S-enriched fraction was performed by omitting β-mercaptoethanol both in the extraction and dialyzing buffers (Thanh et al., 1975). Protein concentration was determined spectrophotometrically at 562 nm using the bicinchoninic acid kit and bovine serum albumin (BSA) as standard.

### SDS-PAGE and gel staining

All one-dimensional electrophoretic runs were performed with the Mini Protean III apparatus (Bio-Rad Laboratories, Segrate, Italy). Proteins (40 μg lane^−1^) were separated by SDS polyacrylamide gel electrophoresis (SDS-PAGE) according to the method of Laemmli (1970). For the glutenin-like enriched fraction, 2 M urea was added to both stacking and resolving gels. The molecular mass standard was the Biomol (Hamburg, Germany) BLUEplus prestained Protein Ladder (10–180 kDa). Gels were fixed at room temperature in 50% methanol: 5% glacial acetic acid for 20 min, then in 50% methanol for 10 min. After fixing, gels were washed twice in deionized water (10 min each) and stained with silver as previously described (Shevchenko et al., 1996) with minor modifications. Protein profiles were densitometrically analyzed using the AIDA software 4.14 (raytest Isotopenmessgeräte GmbH, Straubenhardt, Germany).

### Protein precipitation

For protein precipitation, 4 vol of 20% trichloroacetic acid (TCA) and 0.007% β-mercaptoethanol in cold acetone were added to samples, mixed and kept at −20°C for at least 45 min. Proteins were pelleted by centrifugation at 15,000 × g for 15 min at 4°C and then washed with cold acetone containing β-mercaptoethanol. This step was repeated at least three times and residual acetone was finally removed by air-drying. The last pellet was resuspended with buffer for 2DE analysis (see below) and the protein concentration of samples was determined using a commercial kit (2-D Quant Kit, GE HealthCare, Milan, Italy), performed as described in the instruction manual and using BSA as reference.

### Two-dimensional electrophoresis (2DE)

For 2DE analyses, 11-cm IPG Strips with a 3–10 pH gradient (Bio-Rad) were used in combination with 10% Criterion XT gels (Bio-Rad). Strips were rehydrated in the solubilization buffer (40 mM Tris, 8 M urea, 2 M thiourea, 2% CHAPS, traces of bromophenol blue) to which 18 mM DTT and 20 μl ml^−1^ IPG buffer were added. Samples were dissolved to 1 mg ml^−1^ concentration in the solubilization buffer. Strips were rehydrated overnight in an Immobiline Dry Strip Reswelling Tray covered with a Dry Strip Cover PlusOne (GE HealthCare). Strips were run using a Protean IEF cell (Bio-Rad) through eight different steps:

- From 0 to 300 V for 30 min.
- 300 V for 1 h and 30 min.
- From 300 to 4000 V for 2 h.
- 4000 V for 1 h and 30 min.
- From 4000 to 8000 V for 1 h and 30 min.
- 8000 V until a total of 20,000 Vhr (Volts h^−1^).
- From 8000 to 250 V for 10 min.
- Hold step of 250 V until use of strips.

Strips were stored at −80°C or used immediately. In both cases, they were equilibrated for 15 min in equilibration buffer (50 mM Tris-HCl, pH 8.8 containing 6 M urea, 30% glycerol, 2% SDS, bromophenol blue, 10 mg ml^−1^ dithiothreitol). Proteins were then separated in the second dimension based on a Bis-Tris buffer system (pH 6.4) that uses discontinuous chloride and MES or MOPS ion fronts to form moving boundaries to stack and then separate denatured proteins by size. Molecular weight standards of the Precision series (Bio-Rad) were run in parallel. Gels were stained with Bio-Safe Coomassie blue (Bio-Rad) as described in the instruction protocol.

### Spot analysis

Images of gels were captured using the Fluor-S Multi-Imager (Bio-Rad). The exposure time was 5–7 s for gels stained with Coomassie blue. Analysis of spots in 2DE gels was performed using the Spot Detection Wizard of PDQuest (Bio-Rad) by selecting the weakest protein spot and the larger protein clusters. Subsequently, spot analysis was improved manually by adding unidentified spots and by removing incorrect signals. After creating a Master (virtual) gel, spots were matched to determine qualitative and quantitative differences. Further analysis of spots was done using the Spot and Match set tools. The intensity of protein spots was normalized in relation to the total abundance of effective spots. After normalization and background subtraction, gels from control and treated samples were used to create a match set, which allowed the differential expression analysis between treated and control samples. Spots were considered as up- or down-regulated if their amount changed at least by a factor of 2. All samples were analyzed in duplicate.

### Protein identification by mass spectrometry

Protein identification was performed as previously described (Hellman et al., 1995; Soskić et al., 1999). Spots of interest were manually excised, destained in 2.5 mM ammonium bicarbonate and 50% (v/v) acetonitrile and then dehydrated in acetonitrile. Gel pieces were rehydrated in trypsin solution and in-gel protein digestion was performed by an overnight incubation at 37°C. For MALDI-TOF MS, 1.25 μl of each protein digest was directly spotted onto the MALDI target and air-dried. After drying, 0.75 μl of matrix solution [5 mg ml^−1^ α-cyano-4-hydroxycynnamic acid in 50% (v/v) acetonitrile and 0.5% (v/v) trifluoroacetic acid] was added to samples, which were allowed to dry again. Acquisition of mass spectra was performed using an Ultraflex III MALDI-TOF/TOF mass spectrometer (Bruker Daltonics, Billerica, MA, United States) in reflector positive mode. Spectra were analyzed by Flex Analysis software v. 3.0. Peptide mass fingerprinting (PMF) database searching was carried out in NCBInr and/or Swiss-Prot/TrEMBL databases set for Viridiplantae (Green Plants) using Mascot (Matrix Science Ltd., London, UK, http://www.matrixscience.com) on-line available software. The search settings were as follows: mass tolerance was set at 100 ppm, trypsin as the digestion enzyme with one allowed missed cleavage and oxidation of methionine as a variable modification. In order to accept identifications, the number of matched peptides, the extent of sequence coverage, and the probabilistic score were considered. Peptide digests that did not give unambiguous identifications were subjected to peptide sequencing by tandem mass spectrometry. MS/MS analysis was performed on the Ultraflex III MALDI-TOF/TOF instrument. Two to three PMF peaks showing a high intensity were CID (Collision Induced Dissociation) fragmented using Argon as collision gas, and MALDI-TOF/TOF tandem MS was performed in LIFT mode by software controlled data acquisition. Fragmented ions were analyzed using the Flex Analysis software v. 3.0. The MS/MS database search was carried out in NCBInr and/or Swiss-Prot/TrEMBL databases using the on-line MASCOT MS/MS ion search software. The following parameters were applied for the database search: taxonomy: Viridiplantae (Green Plants), trypsin specificity, one missed cleavage allowed, peptide precursor mass tolerance: ±100 ppm, fragment mass tolerance ±0.6 Da, peptide precursor charge state +1, carbamidomethylation of cysteine as a fixed modification, oxidation of methionine as a possible modification. Protein identification was considered significant based on Mascot ion score, peptide coverage by “b” and “y” ions, and expected value.

### Amino acid analysis

Analysis of free amino acids and amino acids derived from complete hydrolysis of proteins in quinoa flour was performed in triplicate essentially as described by Silvanini et al. (2014) with some modifications. To analyze the content of free amino acids, 0.5 ml dH_2_O and 75 μl trifluoroacetic acid were added to 20 mg of quinoa flour. After mixing, samples were centrifuged at 12,000 g for 5 min at room temperature. Supernatants were dried by Speed-Vac and residues were dissolved in 20 μl of 20 mM HCl. The content of protein-derived amino acids (PAAs) was determined by mixing each flour sample with 6 N HCl and phenol crystal (around 1 mg to avoid oxidation) followed by incubation for 24 h at 110°C. After heating, samples were centrifuged at 4000 rpm for 15 min at room temperature. The supernatant was dried under vacuum and the residue was dissolved in 20 μl of 20 mM HCl. Both free amino acids and those derived from hydrolysed proteins were derivatized according to the AccQ-Tag protocol (Waters, Milford, MA, USA). For HPLC analysis, a C18 AccQ-Tag column (3.9 × 150 mm; Waters, Milford, MA, USA) was used. A gradient elution was performed using a phosphate buffer solution as eluent A and acetonitrile:water 60:40 (v/v) as eluent B. The temperature was set at 37°C; the flow rate was 1 ml min^−1^. The fluorescent detector parameters were set as follows: λex = 250 nm, λem = 395 nm, gain 1, eufs 100. All data are expressed as mg 100 g^−1^ flour.

### Extraction of total proteins

Extraction of total proteins was performed essentially as described by Escudero et al. (2004). After adding 15 ml of double distilled water to an aliquot (50 mg) of the flour and continuous stirring for 30 min at room temperature (RT), the pH was taken to 9.0 with 0.1 N NaOH. After further stirring for 30 min, the homogenate was centrifuged at 4500 × g for 20 min at RT. The supernatant was collected and taken to pH 5.0 with 0.1 N HCl. After stirring for another 20 min and centrifugation at 4500 × g at 4°C, the supernatant was discarded and the pellet re-suspended in 500 μl of 63 mM Tris-HCl, pH 8.0. Protein concentration was determined spectrophotometrically at 562 nm using the bicinchoninic acid kit and BSA as standard.

### Total phenolics and flavonoid contents

The Folin-Ciocalteu (FC) assay was performed according to Singleton and Rossi (1965) with some modifications; 50 μl of protein extract (1 mg protein ml^−1^) were mixed with 250 μl of FC reagent (previously diluted 10-fold with distilled water) and 500 μl distilled water. The reaction mixture was incubated for 1 min at room temperature and then 800 μl of 20% (w/v) Na_2_CO_3_ was added. After incubation at 40°C for 30 min, the absorbance was measured spectrophotometrically at 760 nm (Jasco V-530, Jasco Corporation, Tokyo, Japan). The total polyphenolic content (TPC) was evaluated from a gallic acid standard curve and was expressed as mg gallic acid equivalents (GAE) g^−1^ seed DW. The Prussian blue method (Hagerman and Butler, 1994) was also applied to determine total polyphenols. After adding 12 μl of 0.1 M FeNH_4_(SO_4_)_2_ to 100 μl protein extract (1 mg protein ml^−1^), the mixture was incubated for 20 min at RT. Subsequently, 12 μl of 8 mM K_3_Fe(CN)_6_ were added, and after 5 min at room temperature, the optical density of the mixture was determined at 720 nm. Gallic acid was used as standard to make a calibration curve and data were expressed as mg GAE g^−1^ DW.

The total flavonoid content (TFC) was determined with AlCl_3_ according to Liu et al. (2002) with slight modifications using rutin as standard. The seed protein extract (50 μl) was added to 450 μl of 100% methanol followed by 500 μl of 2% (w/v) AlCl_3_ in methanol. This reaction mixture was incubated for 15 min at room temperature. Finally, the absorbance of the reaction mixture was measured spectrophotometrically at 430 nm. Data were expressed as mg rutin equivalents (RE) g^−1^ DW.

### Radical scavenging activity

Free radical scavenging capacity of the protein extracts was determined by using the ABTS assay performed according to Arnao et al. (2001) with slight modifications. The ABTS^+^ radical cation was generated by oxidizing a 2.0 mM aqueous solution of ABTS with 70 mM K_2_S_2_O_8_ and incubating in the dark for 24 h at room temperature. The reaction mixture contained 1.0 ml of ABTS^+^ (diluted with methanol in order to obtain an absorbance of 0.7 at 734 nm) and different amounts (12.5, 25, 50, 100 μl) of sample solutions (1 mg ml^−1^ total protein or 11S fraction) or Tris buffer for the blank. The absorbance at 734 nm was measured after a reaction time of 20 min. Trolox equivalents per g DW of seeds were calculated using a standard curve prepared with a range of Trolox concentrations (0–30 μM) in order to calculate the Total Antioxidant Activity (TAC) value for the samples.

### Statistical analysis

Two independent experiments were performed. Each experiment consisted of three pots per treatment (0, 100, and 300 mM NaCl), each containing one plant per landrace, and set up according to a randomized block design. Flour preparation, protein extractions, SDS-PAGE, and AIDA analyses were performed at least twice. TPC, TFC, and AA data were performed in triplicate from two separate protein extractions. To determine the overall significances, a two-way factorial analysis of variance (two-way ANOVA) was used with salt treatment and landrace as factors. Mean comparisons were made by applying Tukey's *post-hoc* test using InfoStat software (www.infostat.com.ar). Differences were considered significant at *P* < 0.05.

## Results

### Seed storage protein profiles and spot analysis

Sequential extraction of the different protein fractions and isolation of the major storage proteins were performed on seeds of the three quinoa landraces (R49, VI-I, and VR) grown with/without 100 or 300 mM NaCl. A combination of SDS-PAGE and 2DE was used to, first of all, identify the fractions that were most affected by salt, and second the proteins that were most involved in the salt-induced response. The protein fraction analyzed was the one enriched in albumins/globulins. A general feature was the reduction of these storage proteins, expressed as mg protein mg^−1^ flour, after the 300 mM NaCl treatment. In particular, the albumin/globulin fraction declined by ca. 12, 7, and 15% in R49, VI-I, and VR, respectively (data not shown).

**Figure 1:**
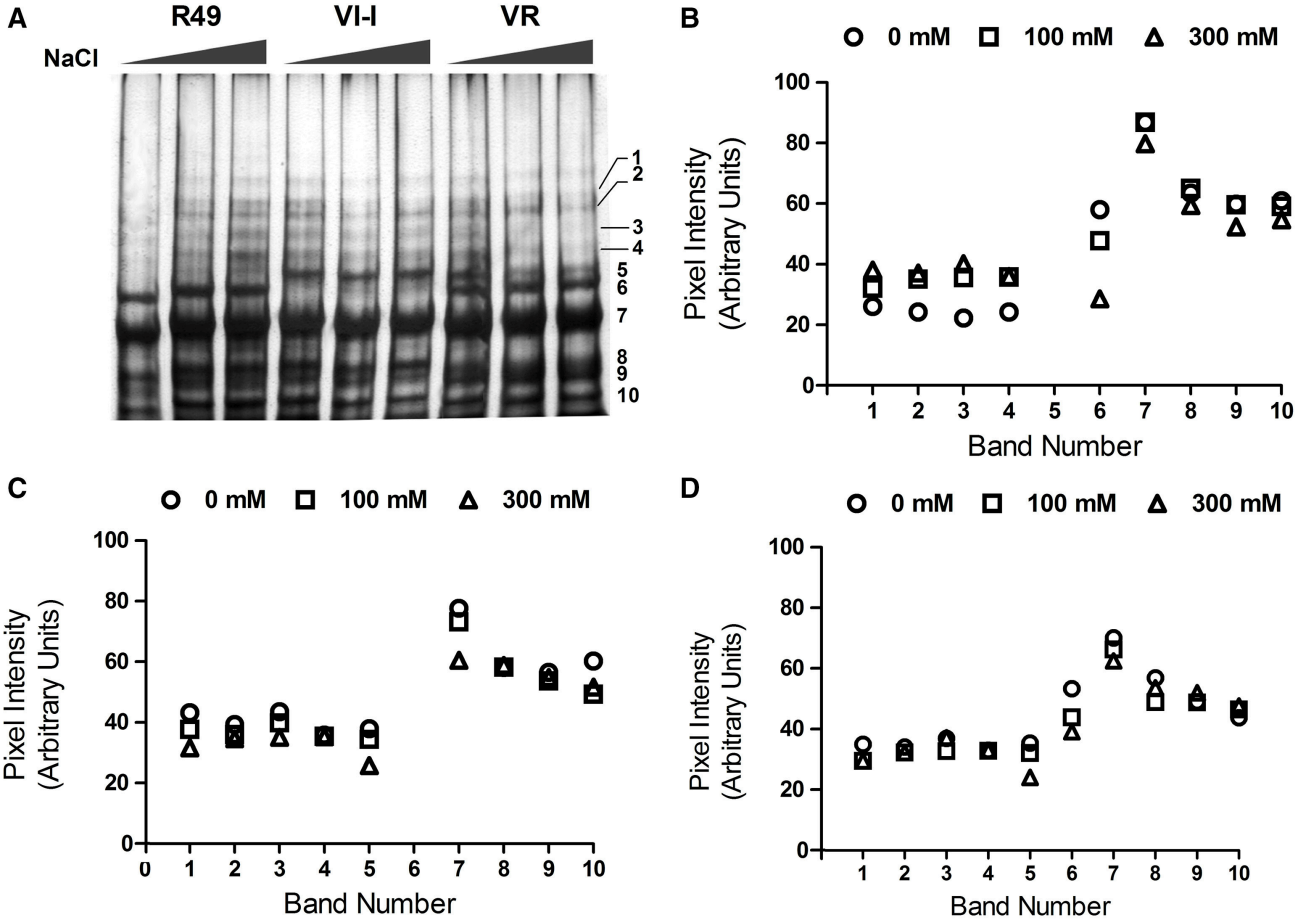
**Representative SDS-PAGE (A) of the albumin/globulin fraction of seeds from plants of three quinoa landraces (R49, VI-I, and VR) irrigated with 0, 100, or 300 mM NaCl**. The 10 major bands identified by the AIDA Image Analyzer software are numbered: 49 (1), 45 (2), 42 (3), 40 (4), 32 (5), 30 (6), 25 (7), 22 (8), 19 (9), and 16 (10) kDa. Intensity of the 10 major bands in control and salt-treated (100 or 300 mM NaCl) samples of the three landraces: R49 **(B)**, VI-I **(C)**, and VR **(D)**.

As shown in Figure 1, the proteins were resolved into distinct bands that spanned a broad range of apparent molecular weights from 15 to >55 kDa. The image analysis software identified 10 major bands of 49 (1), 45 (2), 42 (3), 40 (4), 32 (5), 30 (6), 25 (7), 22 (8), 19 (9), and 16 (10) kDa. Some difference in the band patterns among genotypes was evident: in R49 the 32-kDa (5) band was absent while VI-I seemed to lack the 30-kDa band (6); VR had both the 32- (5); and the 30-kDa (6) bands. Comparing the protein profiles in control and salt-treated seeds by SDS-PAGE revealed that the NaCl treatment induced significant changes in the protein patterns and that the three landraces were differentially affected by salinity. In R49, the profile was very similar in seeds from control and 100 mM NaCl-treated plants; in the 300 mM NaCl treatment, the high molecular weight bands (1–4) appeared to be slightly increased, while the low molecular weight bands (6–10) decreased significantly relative to controls (Figure 1B). Under saline conditions (300 mM NaCl), VI-I showed a decrease in different proteins; the decline was most evident in the low molecular-weight bands except for 8 and 9 (5–10; Figure 1C). Finally, in VR, both salt treatments decreased the intensity of bands 5, 6, and 7 in a concentration-dependent manner (Figure 1D).

**Figure 2:**
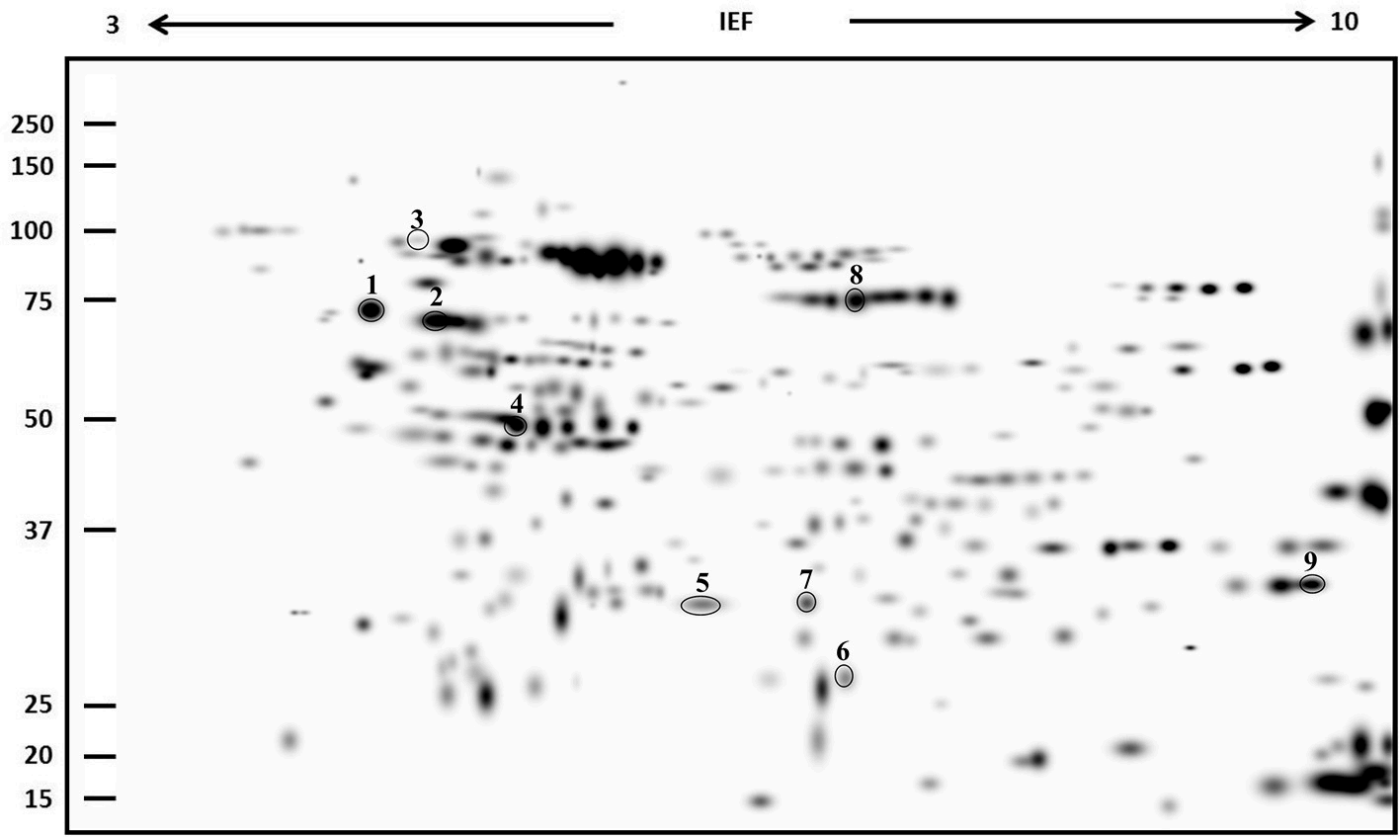
**Master gel obtained from the PDQuest-based comparison of 2DE gels of albumin/globulin fraction samples of VR (control and 300 mM NaCl-treated)**. The virtual image of gel represents all spots as detected in all samples tested. Molecular mass standards are indicated on the left while the pH range is at the top. Equivalent protein contents (200 μg) were loaded in each sample. Spots analyzed by MALDI-TOF are circled and numbered.

**Figure 3:**
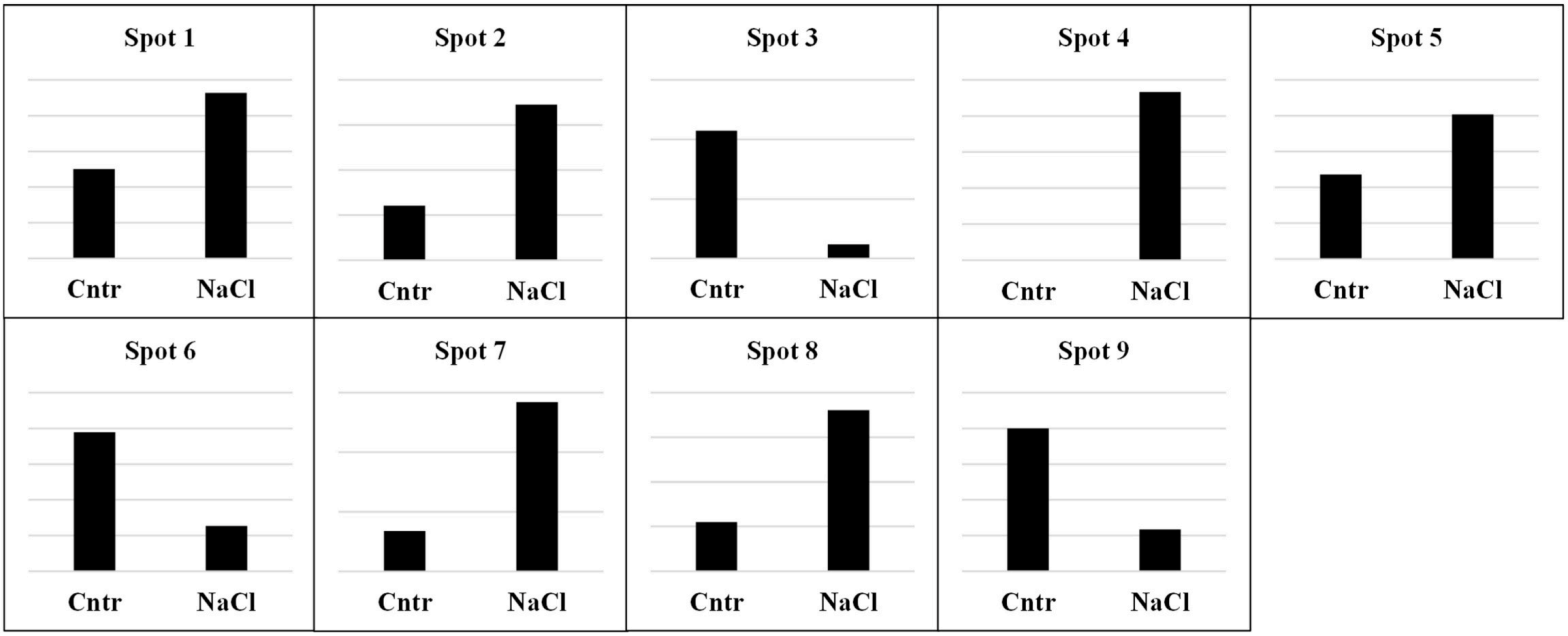
**Relative abundance of individual spots from the albumin/globulin fractions of quinoa seeds harvested from plants grown under control (Cntr) or saline (NaCl) conditions**. Spots are numbered as shown in the master gel (Figure 2). Each spot is presented with the value of its relative quantification. The first column indicates the control sample, while the second column indicates the 300 mM NaCl treatment. Scales are different in the Y-axes of each graph because they were optimized to highlight the different intensity of spots between samples. In all cases, the most intense spot was used as reference for calibrating the scale of Y-axes.

Subsequently, a proteomic profile of the albumin/globulin fraction was performed by 2DE in control and salt-treated samples of landrace VR. This genotype was chosen because, in addition to changes in the SDS-PAGE band pattern, under salinity it also exhibited the highest TPC, TFC, and AA (see below) and the greatest percentage decrease in the albumin/globulin fraction. Two-DE gels revealed significant differences in protein composition between the two samples. Following the analysis of master gels obtained from albumin/globulin fractions, the nine spots whose abundance changed at least two-fold during treatments were selected and processed for identification by MALDI-TOF MS (Figure 2). Spots were numbered arbitrarily and correspond to numbers indicated in the identification list. As shown in Table 1, the spots analyzed by MS revealed specific correspondence to peptides in the protein database. They were clustered into seven functional categories: stress (chaperone/folding), transcription factors, respiration, photosynthesis, storage proteins, metabolism, and cell division. The spot intensities show that significant changes, either increases or decreases, occurred in response to salinity (Figure 3).

**Table 1:** **Proteins identified by MALDI-TOF and/or MALDI-TOF/TOF mass spectrometry during analysis of the albumin/globulin fraction from seeds a quinoa landrace (VR)**.

**Spot**	**Protein name**	**Accession number**	**Principal function**	**pI/MW**	**MASCOT search results**		
					**Score**	**Matched peptides**	**Coverage**
1	Stromal 70 kDa heat shock-related protein, chloroplastic [*Glycine soja*]	gi|734418521	Interacts with newly imported chloroplast proteins to assist in their maturation	4.84 65521	102	13	24
	Heat shock protein, putative [*Ricinus communis*]	gi|255570990	Stress response	5.35 75431	113	15	24
2	GRP-78 [*Spinacia oleracea*]/Luminal-binding protein	gi|3913786	Probably plays a role in facilitating the assembly of multimeric protein complexes inside the ER	5.03 73775	177	21	34
	Luminal-binding protein [*Beta vulgaris* subsp. vulgaris]	gi|731321538	Protein involved in processing ER	4.98 73584	153	19	33
	Luminal-binding protein 5 [*Jatropha curcas*]	gi|802612303	Probably plays a role in facilitating the assembly of multimeric protein complexes inside the ER	5.11 73693	148	19	30
	Endoplasmic reticulum chaperone binding protein [*Lycium chinense*]	gi|749396761	ATP binding	5.08 73343	139	18	30
	Heat shock protein, putative [*Ricinus communis*]	gi|255555659	Stress response	5.17 73464	134	18	28
3	PREDICTED: ETHYLENE INSENSITIVE 3-like 1 protein [*Phoenix dactylifera*]	gi|672174403	Probable transcription factor acting as a positive regulator in the ethylene response pathway. Could bind the primary ethylene response element present in the ETHYLENE-RESPONSE-FACTOR1 promoter	5.08 40252	82	11	29
4	PREDICTED: ATP synthase subunit beta, mitochondrial [*Fragaria vesca* subsp. vesca]	gi|470126069	Produces ATP from ADP in the presence of a proton gradient across the membrane which is generated by electron transport complexes of the respiratory chain	6.01 60119	130	19	46
	Putative ATP synthase beta subunit [*Oryza sativa* Japonica Group]	gi|56784991	Produces ATP from ADP in the presence of a proton gradient across the membrane. The catalytic sites are hosted primarily by the beta subunits	5.33 45937	110	15	48
	PREDICTED: ATP synthase subunit beta, mitochondrial [*Pyrus x bretschneideri*]	gi|694311412	Produces ATP from ADP in the presence of a proton gradient across the membrane. The catalytic sites are hosted primarily by the beta subunits	6.13 60178	108	18	41
	PREDICTED: ATP synthase subunit beta, mitochondrial-like [*Pyrus x bretschneideri*]	gi|694330995	Produces ATP from ADP in the presence of a proton gradient across the membrane. The catalytic sites are hosted primarily by the beta subunits	6.01 60151	108	18	41
5	Plastid movement impaired protein [*Medicago truncatula*]	gi|922369389	Required for the chloroplast avoidance response under high intensity blue light. This avoidance response consists in the relocation of chloroplasts on the anticlinal side of exposed cells. Acts in association with WEB1 to maintain the velocity of chloroplast photo relocation movement via cp-actin filaments regulation	9.94 23523	77	9	42
6	11S seed storage globulin A [*Chenopodium quinoa*]	gi|115343511	Functions in the storage of nutritious substrates	6.88 54034	82	12	21
7	Pantothenate kinase [*Micromonas* sp. RCC299]	gi|255070825	ATP binding and pantothenate kinase activity. Catalysis of the reaction: ATP + pantothenate = ADP + D-4′-phosphopantothenate	5.69 104293	81	11	12
8	Helicase carboxy-terminal domain protein [*Tetrahymena thermophila* SB210]	gi|829168809	ATP binding and helicase activity. Catalysis of the reaction: NTP + H2O = NDP + phosphate, to drive the unwinding of a DNA or RNA helix	7.88 223030	92	23	15
9	Peptidyl-prolyl cis-trans isomerase CYP20-1 [*Amborella trichopoda*]	gi|586718012	PPIases accelerate the folding of proteins. It catalyzes the cis-trans isomerization of proline imidic peptide bonds in oligopeptides. Binds cyclosporin A (CsA). CsA mediates some of its effects via an inhibitory action on PPIase	8.99 22301	77	8	40

*For each spot, number, protein name, organism, accession number, principal functions, theoretical pI, molecular mass (MW), and Mascot search data are shown*.

Three spots (1, 2, and 9) correspond to proteins involved in stress responses or as support in protein folding. In this category, spot 1 corresponds to a stromal chloroplast 70-kDa heat shock-related protein of *Glycine soja*, and to a putative heat shock protein of *Ricinus communis*. Analysis of the spot 2 sequence revealed a correspondence to several proteins with similar function: a GRP-78/luminal-binding protein of *Spinacia oleracea*, a luminal-binding protein of *Beta vulgaris* subsp. *vulgaris*, a luminal-binding protein 5 of *Jatropha curcas*, an endoplasmic reticulum chaperone binding protein of *Lycium chinense*, and a putative heat shock protein of *R. communis*. The intensity of spots 1 and 2 increased after salt treatment. Spot 9 corresponded to peptidyl-prolyl cis-trans isomerase CYP20-1 of *Amborella trichopoda*, a protein involved in enhancing the correct folding of proteins; it decreased in seeds of salt-stressed plants as compared with controls. The second functional category is represented by spot 3, which corresponds to an Ethylene Insensitive3-like 1 protein of *Phoenix dactylifera*, a positive regulator in the ethylene response pathway. Its expression level decreased after treatment with NaCl. A protein involved in respiration was also identified (although only in the NaCl-treated samples). Spot 4 was matched to a mitochondrial beta-subunit of ATP synthase in different organisms (*Fragaria vesca* subsp. *vesca, Oryza sativa* and *Pyrus* × *bretschneideri*). Spot 5 was identified as a plastid movement impaired protein of *Medicago truncatula*; the protein showed an increased level in the NaCl-treated sample. Mass spectrometry analysis revealed that spot 6 corresponded to the 11S seed storage globulin A of *C. quinoa*. The expression level of this protein showed a drastic decrease in samples treated with NaCl. Spot 7 found homology with a pantothenate kinase of *Micromonas sp*. This protein converts pantothenate to phosphopantothenate, using ATP as phosphate donor and was significantly up-regulated under salinity. Finally, spot 8 matched to a helicase protein of *Tetrahymena thermophile*, a protein involved in the unwinding of DNA or RNA helix. The expression level of this protein was drastically increased in samples treated with NaCl.

### Profiles and spot analysis of globulin fractions

**Figure 4:**
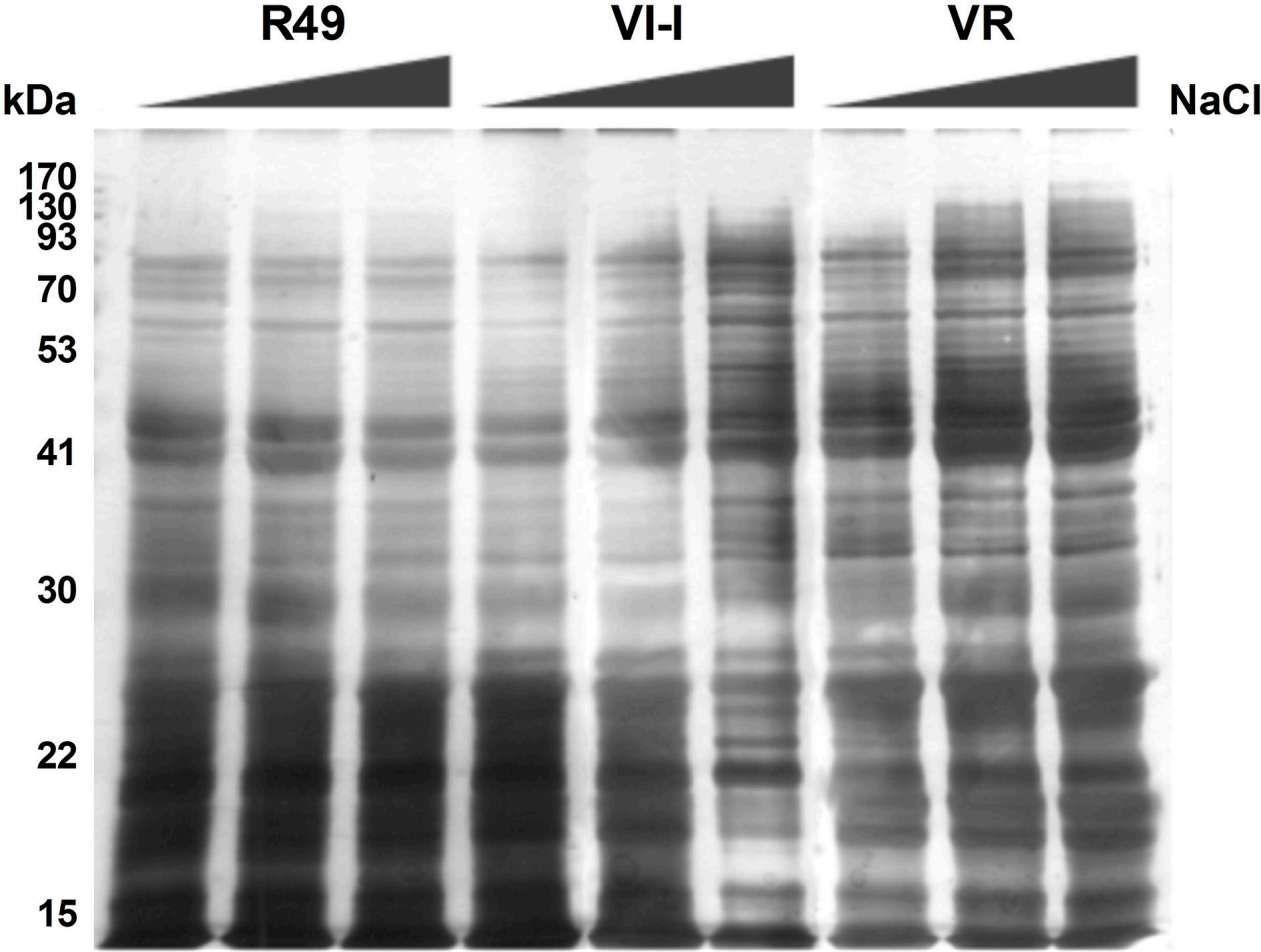
**Representative SDS-PAGE of the crude acid-soluble globulin fraction of seeds of three quinoa landraces (R49, VI-I, and VR) irrigated with 0, 100, or 300 mM NaCl**.

As the albumin/globulin fraction from seed proteins of quinoa was strongly affected by salinity, a further attempt was made to isolate and analyze the crude acid-soluble globulins and the 11S- (chenopodin) enriched fractions by SDS-PAGE. As shown in Figure 4, proteins of the crude acid-soluble globulin fraction were resolved into distinct bands that spanned a broad range of apparent molecular weights from 15 to >90 kDa. Results showed that the protein profiles were represented by eight major and distinct bands, common to all three landraces, with molecular weights of 90, 68, 45, 43, 31, 20, 18, and 15 kDa. Comparing the protein profiles in seeds from control and salt-treated plants revealed that the NaCl treatment did not induce significant changes in the protein composition; however, seeds of plants grown under 300 mM NaCl showed a general reduction in these storage proteins. In particular, the crude acid-soluble globulin fraction declined by 22.5, 13.9, and 25.3% in R49, VI-I, and VR, respectively.

**Figure 5:**
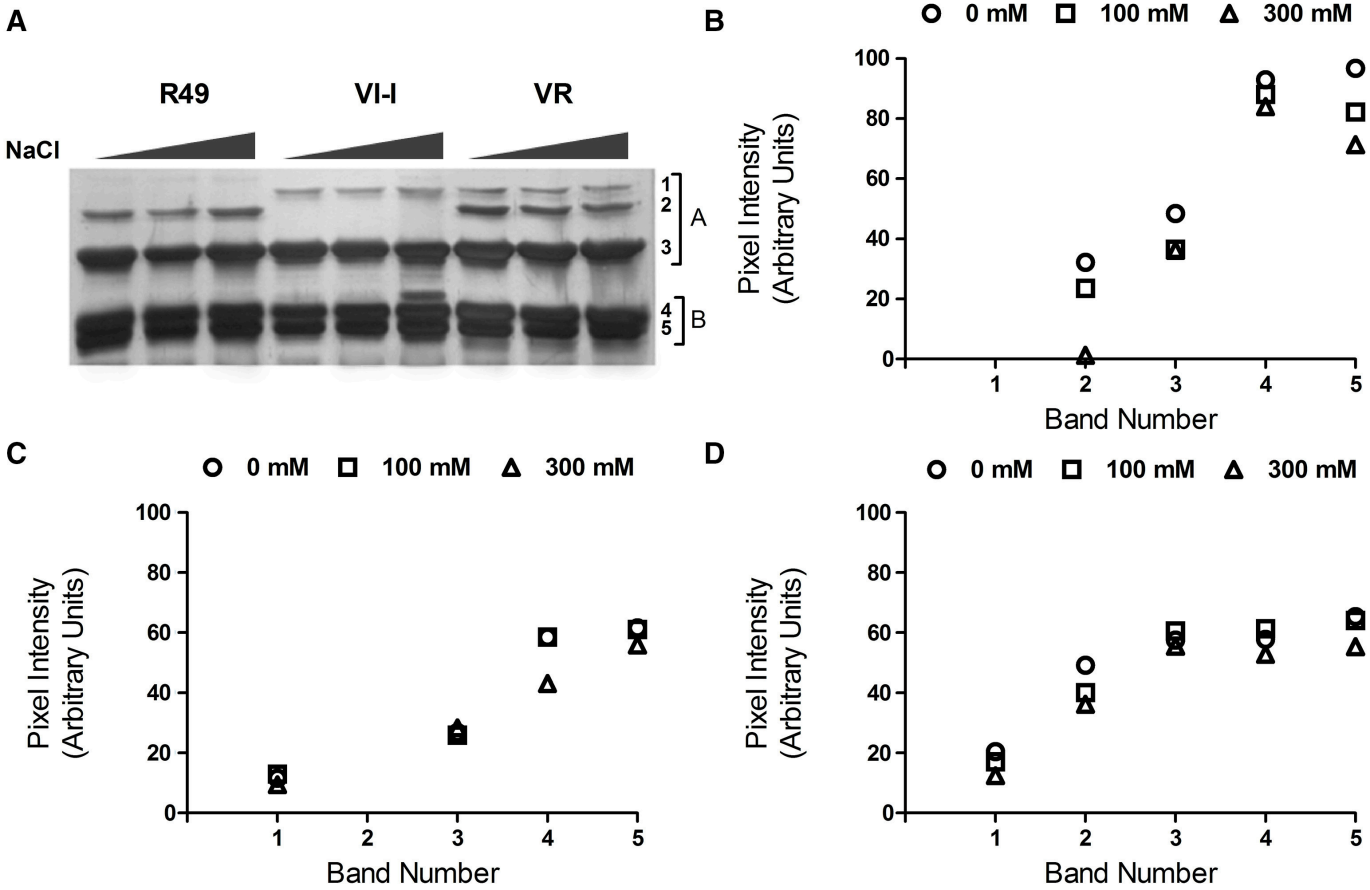
**Representative SDS-PAGE of the 11S (chenopodin)-enriched fraction from seeds of three quinoa landraces (R49, VI-I, and VR) irrigated with 0, 100, or 300 mM NaCl (A)**. The five major bands identified by AIDA Image Analyzer software are labeled with numbers: 42 (1), 40 (2), 32 (3), 24 (4), and 22 (5) kDa. The intensity of the five major bands is shown for control and salt-treated (100 or 300 mM NaCl) landraces: R49 **(B)**, VI-I **(C)**, and VR **(D)**.

As shown by 2DE followed by mass spectrometry of the albumin/globulin fraction, a general reduction of chenopodin, the major 11S storage protein, was also evident (Figure 5). Proteins of the 11S-enriched fractions were resolved into distinct bands giving the typical electrophoretic pattern of chenopodin, in which subunits A (32–42 kDa) and B (22–23 kDa) were clearly evident (Figure 5A). By image analysis, five major bands of 42 (1), 40 (2), 32 (3), 24 (4), and 22 (5) kDa were identified. As already reported for the albumin/globulin fraction, protein band patterns showed some differences among genotypes: the 42-kDa (1) band was absent in R49, VI-I did not present the 40-kDa (2) band and VR had both. Comparing the protein profiles in control and salt-treated seeds by SDS-PAGE revealed that the NaCl treatment induced a significant reduction in the 11S-enriched fraction and that the three landraces were differentially affected by salinity (Figures 5B–D).

**Figure 6:**
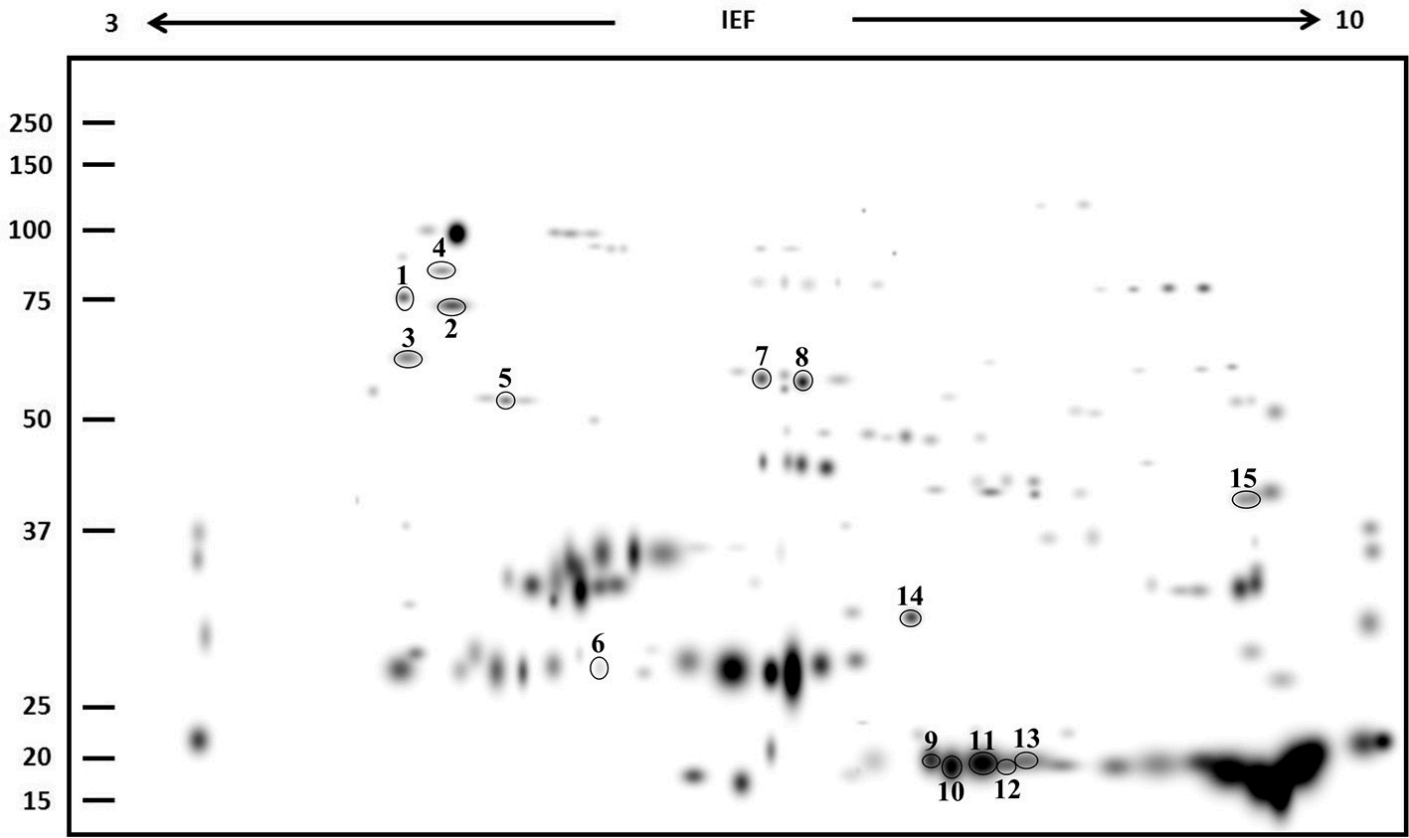
**Master gel obtained from the PDQuest-based comparison of 2DE gels of the 11S fraction from control and 300 mM NaCl-treated samples**. The virtual gel image represents all spots as detected in all samples. Molecular mass standards are indicated on the left while the pH range is at the top. Equivalent protein contents (200 μg) were loaded in each sample. Spots analyzed by MALDI-TOF are circled and numbered.

**Figure 7:**
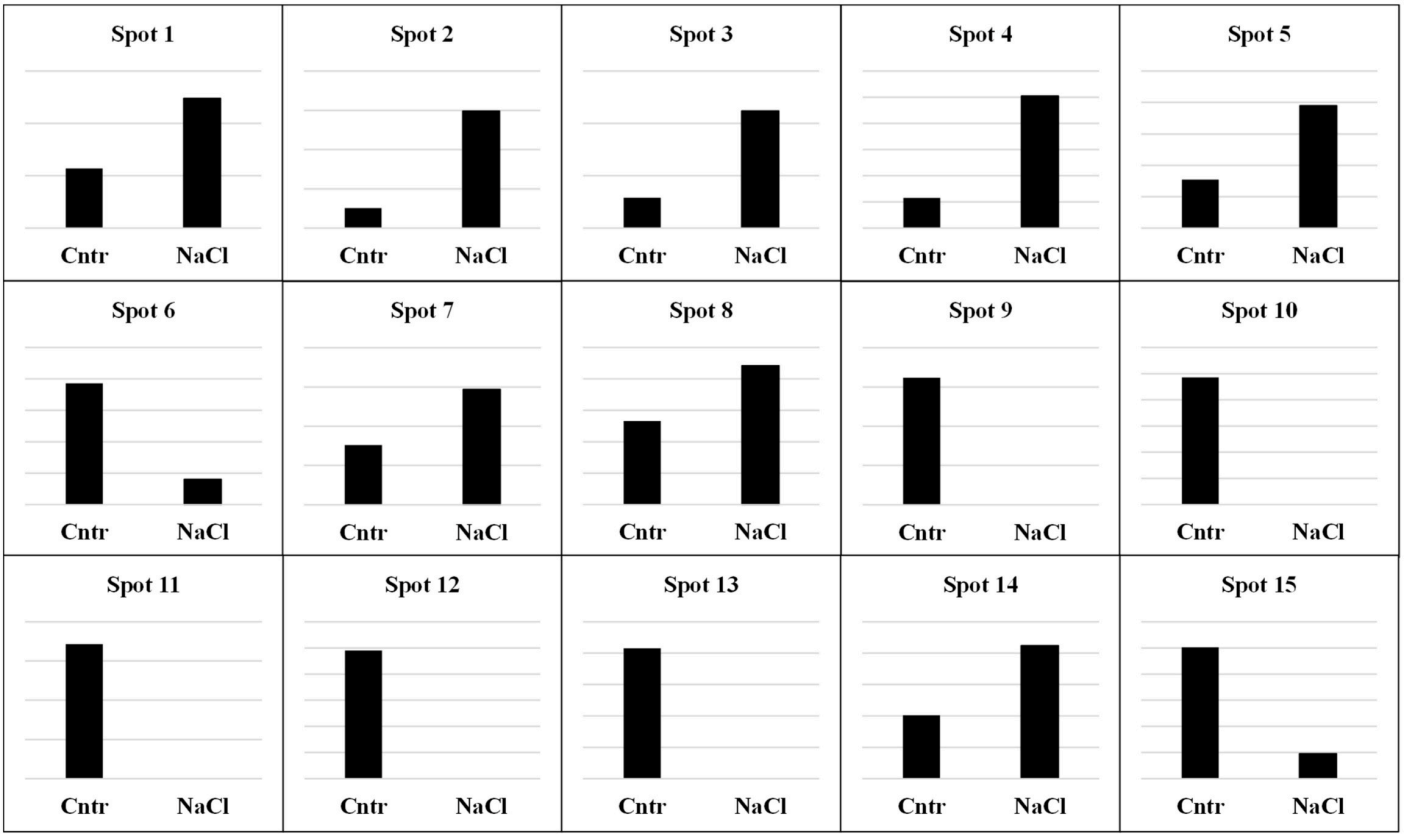
**Relative abundance of individual spots in the 11S fraction of quinoa seeds harvested from plants grown under control (Cntr) or saline (NaCl) conditions**. Spots are numbered as shown in the master gel (Figure 6). Each spot is presented with the value of its relative quantification. Scales are different in the Y-axes of each graph because they were optimized to highlight the different intensity of spots between samples. In all cases, the most intense spot was used as reference for calibrating the scale of Y-axes.

As previously done for the albumin/globulin fraction, a proteomic profile of the 11S fraction was obtained by 2DE. Only landrace VR was analyzed because, as outlined above, all of the five main bands were affected by salt treatment. The comparison between control and salt-treated samples revealed remarkable differences in protein composition (Figure 6). Fifteen spots whose expression levels changed significantly were further analyzed by mass spectrometry. They revealed a specific correspondence to proteins found in databases (Table 2). As in the case of the albumin/globulin fraction, the proteins were clustered into five main functional categories: stress (chaperone/folding), storage proteins, photosynthesis, respiration, and ion transport. The spot intensities in seeds of plants grown under non-saline or saline (300 mM NaCl) conditions are represented in Figure 7. Three different proteins (spots 1, 2, and 4) are involved in stress (chaperone/folding). Two were identified as hypothetical and predicted proteins; however, subsequent BLAST analysis revealed that they corresponded to heat shock 70-kDa protein 6, chloroplastic-like of *Gossypium raimondii* and heat shock cognate 70-kDa protein 1 of *Aegilops tauschii*, respectively. The third protein was identified as a heat shock protein 90 of *Nicotiana tabacum*. In all cases, salt stress increased the accumulation of these proteins compared to controls.

**Table 2:** **Proteins identified by MALDI-TOF and/or MALDI-TOF/TOF mass spectrometry during analysis of the 11S fraction of quinoa landrace VR seed**.

**Spot**	**Protein name/Organism**	**Accession number**	**Principal function**	**BLAST**	**pI/MW**	**MASCOT search results**			
						**Score**	**Matched peptides**		**Coverage**
1	Hypothetical protein B456_006G232400 [*Gossypium raimondii*]	gi|763770793	Preventing aggregation, assisting refolding, protein import and translocation, signal transduction, and transcriptional activation	Heat shock 70 kDa protein 6, chloroplastic-like [*Gossypium raimondii*]	5.45 63324	135	12		27
2	Predicted protein [*Hordeum vulgare* subsp. vulgare]	gi|326506132	Preventing aggregation, assisting refolding, protein import, and translocation, signal transduction, and transcriptional activation	Heat shock cognate 70 kDa protein 1 [*Aegilops tauschii*]	5.07 71476	117	16		28
3	RuBisCO large subunit-binding protein subunit alpha, chloroplastic [*Brassica napus*]	gi|1351030	RuBP carboxylase/oxygenase (RubisCO) catalyzing the actual primary CO2 fixation reaction		4.84 57714	115	14		30
4	Heat shock protein 90 [*Nicotiana tabacum*]	gi|46093890	Facilitating maturation of signaling molecules, genetic buffering		4.95 80358	102	10		15
5	ATP synthase subunit beta, mitochondrial [*Prunus mume*]	gi|645270102	Produces ATP from ADP in the presence of a proton gradient across the membrane which is generated by electron transport complexes of the respiratory chain		5.20 45989	118	11		41
6	11S seed storage globulin [*Chenopodium quinoa*]	gi|45510877	Seed storage			FYLAGKPQQEHSR			
7	PREDICTED: legumin B-like [*Beta vulgaris* subsp. vulgaris]	gi|731320841	Functions in the storage of nutritious substrates	ms/ms		SFFLAGNPQGR			
8	PREDICTED: legumin B-like [*Beta vulgaris* subsp. vulgaris]	gi|731320841	Seed storage			SFFLAGNPQGR			
9	11S seed storage globulin A or B [*Chenopodium quinoa*] C-fragment	gi|115343511 or gi|115343513	Functions in the storage of nutritious substrates		6.88 54034 or 6.58 53942	82 or 73	15 or 14		32 or 31
10	11S seed storage globulin A or B [*Chenopodium quinoa*] C-fragment	gi|115343511 or gi|115343513	Functions in the storage of nutritious substrates		6.88 54034 or 6.58 53942	79 or 70	13 or 12		28 or 27
11	11S seed storage globulin A or B [*Chenopodium quinoa*] C-fragment	gi|115343511 or gi|115343513	Functions in the storage of nutritious substrates		6.88 54034 or 6.58 53942	79 or 70	13 or 12		28 or 27
12	11S seed storage globulin A or B [*Chenopodium quinoa*] C-fragment	gi|115343511 or gi|115343513	Functions in the storage of nutritious substrates		6.88 54034 or 6.58 53942	88 or 78	14 or 13		32 or 31
13	11S seed storage globulin A or B [*Chenopodium quinoa*] C-fragment	gi|115343511 or gi|115343513	Functions in the storage of nutritious substrates		6.88 54034 or 6.58 53942	85 or 75	14 or 13		28 or 27
14	PREDICTED: cation/H(+) antiporter 6B [*Camelina sativa*]	gi|727579738	Monovalent cation: proton antiporter activity; potassium ion transport, sodium ion transport			DSIILGIIMGTK			
15	11S seed storage globulin A or B [*Chenopodium quinoa*] C-fragment	gi|115343511 or gi|115343513	Functions in the storage of nutritious substrates		6.88 54034 or 6.58 53942	130 or 118		13	31 or 29

*For each spot, number, protein name, organism, accession number, principal functions, theoretical pI, molecular mass (MW), and Mascot search data are shown*.

For the storage category, nine spots corresponding to the 11S seed storage globulin of *C. quinoa* and a predicted-legumin B-like of *B. vulgaris subsp. vulgaris* were identified. The expression levels of these proteins changed significantly under salinity. In the case of 11S globulin, the salt-treated sample showed a significant down-regulation or a complete disappearance of spots 6, 9, 10, 11, 12, and 13. On the other hand, the expression level of spots 7 and 8 increased in salt-treated samples compared to controls. Spot 3 corresponds to a chloroplastic RuBisCO large subunit-binding protein, subunit alpha of *Brassica napus*. This photosynthetic protein increased after salt treatment. For the respiration category, spot 5 had a significant correspondence to mitochondrial ATP synthase subunit beta of *Prunus mume* and its intensity increased during stress. The ion transport category was represented by spot 14, which corresponds to a predicted cation/H^(+)^ antiporter 6B of *Camelina sativa* whose intensity increased during stress.

### Prolamin-like proteins

**Figure 8:**
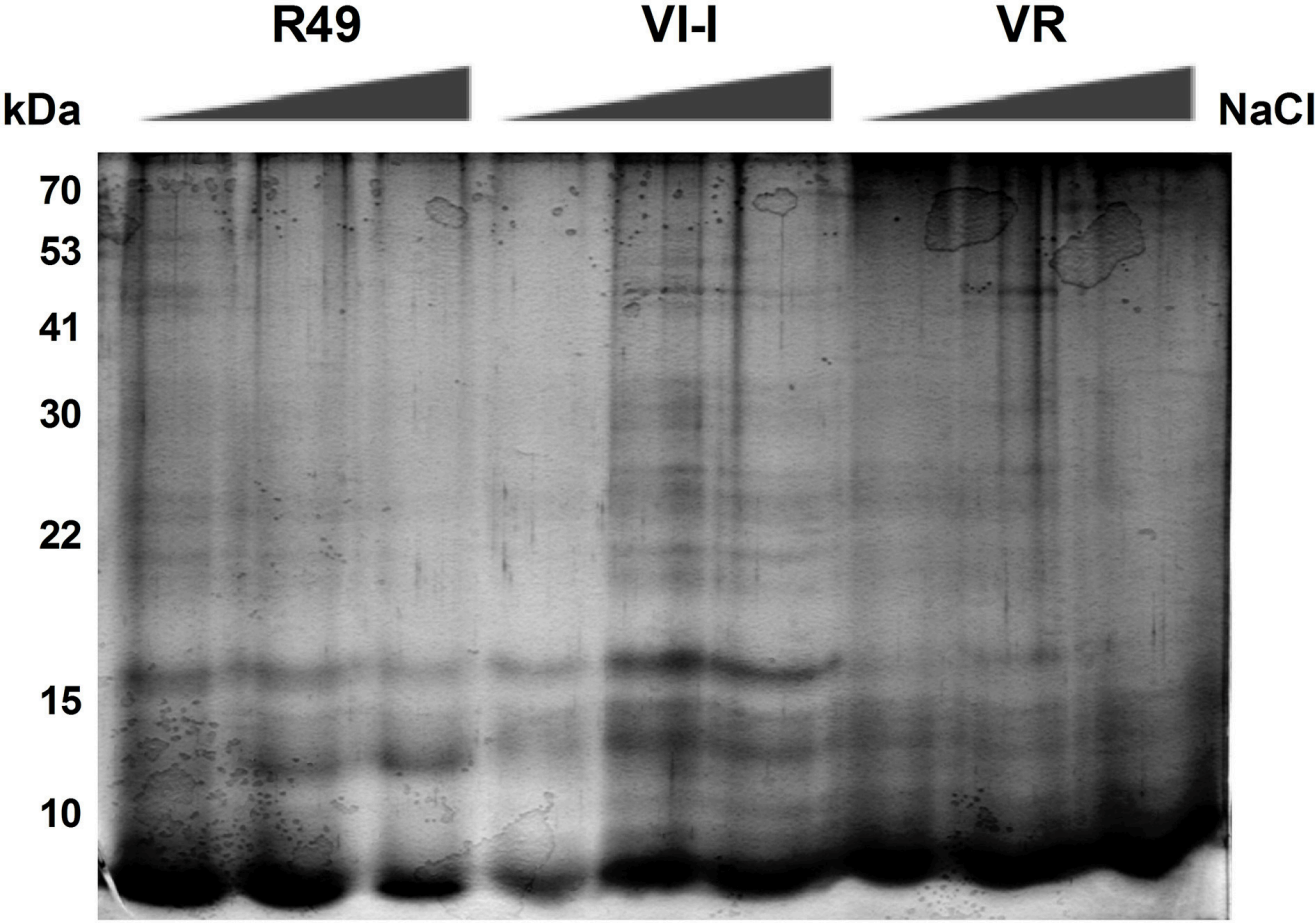
**Representative SDS-PAGE of the ethanol-soluble prolamin-like proteins**.

Detecting and separating quinoa prolamin-like proteins was quite problematic because these polypeptides are scarcely soluble. The ethanol-soluble prolamin-like proteins were scarce and consisted of low-molecular weight polypeptides (Figure 8). Comparing the protein profiles in control and salt-treated seeds by SDS-PAGE revealed that the NaCl treatment did not induce changes in the pattern of proteins possibly related to gluten.

### Amino acid profiles

**Figure 9:**
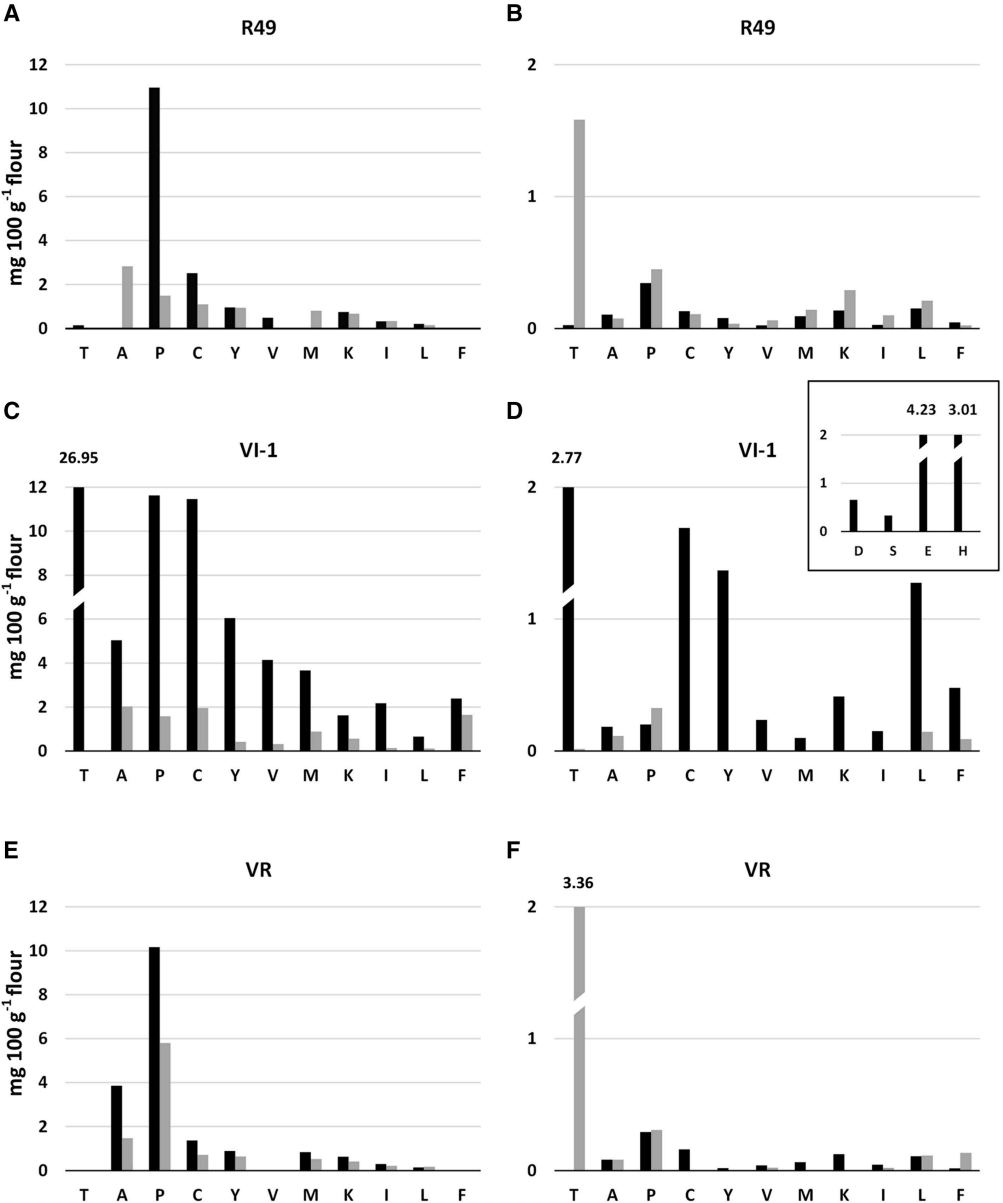
**Contents (mg amino acid 100 g^**−1**^ flour) of protein-derived amino acids (A,C,E) and free amino acids (B,D,F) in seeds of control (black) and salt-treated (gray) landraces R49, VI-1, and VR**. D, Aspartic acid; S, Serine; E, Glutamic acid; G, Glycine; H, Histidine; R, Arginine; T, Threonine; A, Alanine; P, Proline; C, Cysteine; Y, Tyrosine; V, Valine; M, Methionine; K, Lysine; I, Isoleucine; L, Leucine; F, Phenylalanine.

The HPLC analysis of free amino acids and of amino acids derived from the complete hydrolysis of proteins (PAAs) from control and salt-treated seeds of quinoa revealed that some (Asp, Ser, Glu, Gly) were below the limit of detection in most samples and were present only as free amino acids in VI-1 controls (Figure 9D, inset). Overall, the PAA profile was quite different from that of the free amino acids, both quantitatively (PAAs were much more abundant) and qualitatively (Figure 9).

In seeds of control plants, the profile derived from protein hydrolysis showed that Pro was the most abundant PAA in R49 and VR, followed by Cys and Tyr in R49, and by Ala, Cys, Tyr and Met in VR; in VI-1, Pro content was comparable to that of the other landraces, but the most abundant amino acid was Thr (several fold higher than the other amino acids, Figures 9A,C,E). In control plants of VI-1, by far the most abundant essential free amino acids were His and Thr, followed by essential and non-essential amino acids Cys, Tyr, Leu, Glu; in R49 and VR these amino acids were much less abundant or not detectable (Figures 9B,D,F). Pro was the most represented free amino acid in these two landraces followed by Cys. Free Lys content was also ca. three-fold higher in VI-1 than in the other landraces.

Salt differentially affected the amino acid composition of the three landraces. Again, the PAA profile differed substantially from that of free amino acids also in salt-treated samples (Figures 9A,C,E). The non-essential PAAs Ala, Pro, and Cys decreased strongly in all landraces relative to controls, except for Ala in R49, which increased. In R49 there were no changes in some essential amino acids (Lys, Ile, Phe) while other essential amino acids (Thr, Val) declined. Met was the only essential PAA that increased in response to salinity, but only in R49. In VR, all essential PAAs either decreased (Met, Lys, Ile) or remained unchanged; only Leu increased slightly (30% above control value). The strongest overall salt-induced reduction in amino acids was observed in VI-1.

It is noteworthy that in R49 free Thr was induced 60-fold by salinity; other free essential amino acids, namely Val, Lys, and Ile were also significantly enhanced (ca. 2.6−, 2.1−, 3.6-fold) while Leu increased by 25% (Figures 9B,D,E). By contrast, in VR and VI-1, all free essential amino acids were down-regulated, with the exception of Phe in VR, which increased by ca. 7.5-fold. Amongst the non-essential amino acids Ala, Pro, and Cys, there were no or very limited reductions (20–40%) in all three landraces and Pro even increased by 30 and 60% in R49 and VI-1, respectively. In VI-1, most free amino acids were undetectable in salt-treated samples showing a similar trend to the PAAs; in this landrace, Thr disappeared under salinity, whereas in R49 and VR the opposite occurred.

### Total phenolic and flavonoid content, and antioxidant activity

**Figure 10:**
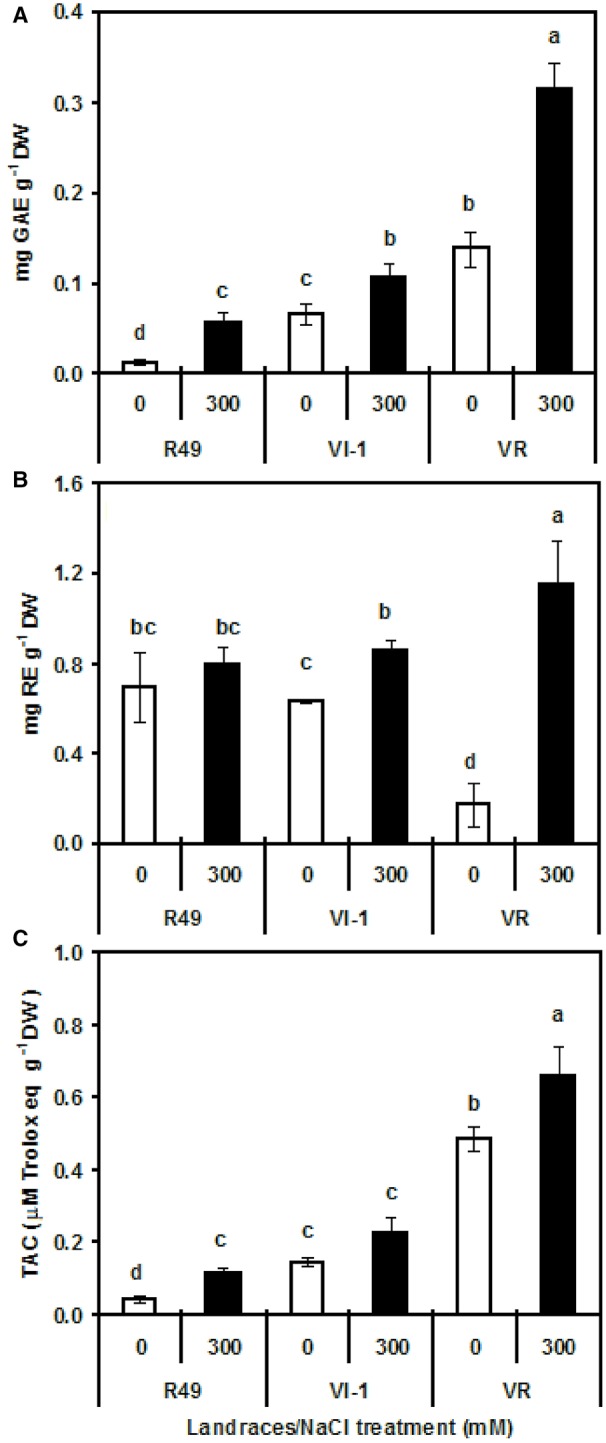
**Total polyphenols as assayed by the Folin-Ciocalteu method (A), total flavonoids (B), and total antioxidant activity (TAC, C) in protein extracts of quinoa seeds harvested from plants grown under control (0) or saline (300 mM NaCl) conditions**.

In the total proteins extracted from quinoa seeds, there was a genotype-dependent and salt-dependent variation in TPC as determined by the FC assay (Figure 10A). Under non-saline conditions, VR had the highest values followed by VI-1and R49. In all three landraces, seeds from plants grown with 300 mM NaCl had higher TPC than those grown without NaCl. The strongest increase (3.5-fold) was observed in R49 while in VR and VI-1 the increase was lower (ca. 60–70% above control levels). Using the Prussian blue method, polyphenols were below detection limit in R49 and VI-1 control and treated seeds; in VR controls, polyphenols were confirmed to be substantially more abundant than in the other two landraces (0.17 ± 0.007 GAE mg^−1^ DW) and dramatically enhanced (five-fold) under salinity (data not shown). Flavonoid concentrations were similar in R49 and VI-1 but lower in VR; however, salinity enhanced TFC only in VR (Figure 10B). The radical scavenging capacity of the seed protein extracts assayed with the ABTS method indicated that TAC was significantly different between landraces, with VR exhibiting higher values as compared with R49 and VI-1. It was slightly but significantly higher in seeds from salt-treated plants of R49 and VR as compared with controls (Figure 10C). The 11S globulin-enriched fraction, extracted without using β-mercaptoethanol, also revealed some ABTS radical scavenging capacity. Values were much lower (0.005–0.006 mM Trolox equivalent g^−1^ DW) than those of the total protein extract and did not change significantly in salt-treated vs. control samples (data not shown).

## Discussion

Quinoa seeds are regarded as one of the most nutritionally well-balanced plant foods under cultivation, especially for their protein content and excellent balance between carbohydrates, lipids, and proteins. The increasing interest in quinoa as a crop is also centered on its remarkable adaptability to harsh environmental conditions such as low water content and high alkalinity of saline soils. Soil salinity is, in fact, among the major factors limiting crop yield and productivity worldwide and is expected to increase in the future. Several comparative studies have shown that the extent to which these and other parameters are affected by salinity in quinoa is strongly genotype-dependent (Gómez-Pando et al., 2010; Adolf et al., 2012).

In the framework of our current investigations on salinity tolerance in quinoa, we have measured growth, yield, germination capacity, leaf TPC, and seed quality in the three Chilean landraces R49, VI-I, and VR exposed to two levels of salinity (Ruiz et al., 2016a). In the present work, we aimed to delve further into the effects of salt treatment on the seed proteome and on the polyphenol content, and AA of seed extracts. The PC of *Amaranthus cruentus* seeds were reported to have improved nutritional quality as compared with flour through increased content of factors that directly or indirectly influence lipid metabolism and enhance antioxidant protection (Escudero et al., 2011). The AA of PCs obtained through different pilot-scale industrial processes from *A. mantegazzianus* seeds was recently evaluated in order to optimize production for the food industry (Castel et al., 2014).

### Proteomic changes

Preliminary analyses were conducted on the three different Chilean landraces in order to unveil genotype-specific responses. In fact, seed protein electrophoresis has been utilized in taxonomy for the explanation of the origin and evolution of a number of cultivated plants (Ahmad and Slinkard, 1992; Jha and Ohri, 1996; Nath et al., 1997; Ghafoor et al., 2002). Seed protein profiles of 40 wild and cultivated taxa of *Chenopodium* were congruent with taxonomic position, crossability relationships, and other biochemical characters, confirming SDS-PAGE as a powerful tool in solving taxonomic problems (Bhargava et al., 2005). More recently, Džunková and co-workers showed that both classical SDS-PAGE and chip electrophoresis analysis of seed storage proteins were useful methods for studying amaranth taxonomy in order to assess differences among different species and even among accessions (Džunková et al., 2011). They suggested that specific fractions, including albumin- and glutelin-enriched fractions, instead of whole extracts, could better reveal differences among landraces. In the present study, the best candidates for SDS-PAGE-mediated polymorphism analysis were the most abundant seed storage proteins, i.e., the albumin/globulin fraction. In fact, results confirm a high polymorphism in seed storage proteins both in the position and in the intensity of some bands. The two most representative genotype-specific bands of this fraction were the 30- and 32-kDa bands.

Because of their low concentrations, prolamins were not suitable for this type of analysis, both in quinoa, herein analyzed, and amaranth and Džunková et al. (2011). While the globulins of amaranth did not differ in band intensity and position (Džunková et al., 2011), we showed that chenopodin, the 11S globulin of quinoa, displayed a high polymorphism in the three landraces analyzed, with genotype-specific protein bands of 40- and 42-kDa. Balzotti et al. (2008) reported the phylogenetic relationships between quinoa and 49 other species by using the coding DNA sequence for the well-conserved 11S basic subunit. On the basis of amino acid alignments, more than 74% sequence identity between amaranth and quinoa was revealed.

To better understand the effects of salt stress on quinoa seeds, we further investigated the composition of storage proteins after irrigation with 300 mM NaCl as compared with untreated controls. Both quantitative and qualitative differences in protein expression as a result of salt treatment were found. The albumin/globulin fraction, and in particular the 11S globulin, was the most affected fraction in terms of reduced percentage content as well as altered profile, both in SDS-PAGE and 2DE. Proteomic analysis revealed increases in the expression of proteins known to be involved directly in ER lumenal protein folding and in the assembly of proteins (Shewry et al., 1995), thus indirectly in the response to cellular stresses. Thus, despite a general decrease in protein content, up-regulation of some specific proteins, such as proteins involved in specific and fundamental cellular processes (photosynthesis and metabolism) as well as in stress adaptation, was observed by analyzing in detail the selected landrace (VR).

An interesting finding was that the expression level of heat shock-related protein (HSP70 and HSP90) and of a GRP-78/Luminal-binding protein (an endoplasmic reticulum chaperone involved in response to different types of stress) increased after salt treatment. Abiotic stresses, such as salinity, usually cause protein dysfunction. Maintaining proteins in their functional conformations thereby preventing the aggregation of non-native proteins is particularly important for cell survival under stress. Heat-shock proteins (HSPs)/chaperones are responsible for protein folding, assembly, translocation, and degradation in many normal cellular processes; they also stabilize proteins and membranes, and can assist in protein refolding under stress conditions (Wang et al., 2004). Data presented in this paper are supported by previous findings showing that at late developmental stages, when seeds undergo dehydration, LEA proteins, also regarded as chaperonins, are synthesized as part of the developmental program. Moreover, salinity triggers an abscisic acid (ABA)-mediated response that induces dehydrin accumulation in embryo cells (especially in the nucleus). This could imply a role for dehydrins as protective molecules for DNA when the cells are undergoing the normal dehydration process. Under salinity, however, dehydration could be drastically exacerbated, thereby favoring chaperonin accumulation (Burrieza et al., 2012). In *Arabidopsis thaliana* cell suspension cultures, the expression of HSP70 also increased under salt stress (Ndimba et al., 2005). Worthy of notice was the reduced content of a member of the peptidyl-prolyl cis-trans isomerase family, also called cyclophilins, known to assist protein folding by accelerating the isomerization of Xaa-Pro bonds, which is a rate-limiting step in the folding process of some proteins. This could be explained by the general decrease of proteins, including the substrates of cyclophilins.

Other proteins of quinoa seeds that responded to salinity are involved in the photosynthetic machinery, i.e., RuBisCO and plastid movement impaired protein. The RuBisCO enzyme is sensitive to salt stress as revealed by enhanced expression in leaves of salt-treated *Triticum durum* (Caruso et al., 2008). In developing embryos of *B. napus* L. (oilseed rape), RuBisCo acts without the Calvin cycle. This improves the carbon efficiency of developing green seeds in a metabolic context able to increase the efficiency of carbon use during the formation of oil (Schwender et al., 2004). Although photosynthesis is usually negatively affected by salinity, the enhanced expression of RuBisCO without the Calvin cycle could be involved in plant adaptation to salt stress at the seed level.

The Plastid Movement Impaired protein is required for regulation of chloroplast position in cells in order to reduce their avoidance. Unfortunately, it is difficult to assign a specific function in seed tissues to this protein directly involved in movement of plastids in response to light and normally expressed in leaves. We can only speculate that salt stress and, more generally, abiotic stress cause changes in the expression level of this protein. Salt stress also seemed to perturb proteins involved in respiration. In fact, the expression of mitochondrial ATP synthase was up-regulated in seeds of salt-treated quinoa plants. Mitochondrial ATP synthase is a key enzyme in plant metabolism and upregulation of its activity might be required in response to stressful conditions such salt stress. Increased production of ATP might fuel all the metabolic pathways generally involved in plant homeostasis. In addition, it has been proposed that increased activity of ATP synthase creates the driving force for Na^+^ transport by the Salt Overly Sensitive1 (SOS1) membrane-localized proteins, whose activity is currently regarded as a strong basis for salt tolerance in plants (Zhu, 2003). Over-expression of an *ATP synthase* gene in Arabidopsis suspension-cultured cells increased resistance to salt, drought, oxidative, and cold stresses (Zhang et al., 2008). Up-regulation of *SOS1* gene expression was also reported for Chilean landraces of quinoa grown on saline media (Ruiz-Carrasco et al., 2011).

Salinity also affected three other main protein categories. Increased expression levels were observed for ion transport proteins (e.g., a cation/H^+^ antiporter), and for pantothenate kinase, and helicase carboxy-terminal domain protein involved in metabolism and cell division, respectively. The over-expression of these proteins during stress, and more specifically salt stress, has been reported in several studies. In particular, expression of the cation antiporter was found to increase in salt-treated tomato plants; the protein plays an important role in adaptation to salinity by improving K^+^ accumulation (Rodriguez-Rosales et al., 2008). Pantothenate kinase activity plays a critical role in regulating intracellular coenzyme A (CoA) levels in bacteria and animals. In plants, pantothenate kinase activity was first reported in stroma of spinach chloroplasts, but little is known about the role of this enzyme (Tilton et al., 2006). Pantothenate, also known as vitamin B5 or “anti-stress vitamin,” is part of the water-soluble B vitamin group. It is the key precursor of CoA and acyl carrier protein, which are essential co-factors for many metabolic enzymes (Raman and Rathinasabapathi, 2004). It was suggested that an increased level of CoA, mediated by an increased activity of pantothenate kinase, may be responsible for improved plant growth and stress resistance (Rubio et al., 2008). The main function of helicase is to bind ATP and to catalyze the reaction that drives the unwinding of DNA or RNA helix. Over-expression of this protein is one of the first cellular responses to stress conditions. Thus, pea DNA helicase was stimulated by salinity and cold stress in both shoots and roots (Vashisht et al., 2005). Moreover, DNA helicase 45 mRNA was induced in pea seedlings in response to high concentrations of salt and overexpression of this protein in transgenic tobacco plants conferred salinity tolerance, suggesting an important target pathway for manipulating stress tolerance in crop plants (Sanan-Mishra et al., 2005).

Ethylene Insensitive 3-like 1 protein is probably a transcription factor acting as positive regulator in the ethylene signaling pathway. The expression of this protein decreased in salt-treated quinoa seeds. Ethylene plays important roles in multiple aspects of plant growth and development and its biosynthesis is induced by many stresses. However, its role in salt stress is uncertain. Cao et al. (2007) reported that alteration of ethylene signaling in *Arabidopsis* mutants affected salt-stress responses. El-Iklil and coworkers showed that lower ethylene production was associated with salt tolerance in tomato treated with high salt concentrations (El-Iklil et al., 2000), whereas higher ethylene production has been suggested as an indicator for salt tolerance in rice seeds (Khan et al., 1987). No information is available on the hormonal responses to abiotic stress in quinoa, except for one report on increased ABA levels under drought (Jacobsen et al., 2009).

The category exhibiting the greatest variation in expression levels under salinity was the functional category of seed storage proteins. Storage proteins accumulate in developing seeds as a source of nitrogen, carbon, sulfur, and amino acids for use in germination and growth of developing seedlings. Globulins generally fall into two major groups based on these coefficients: the 7-8S vicilin type and the 11-12S legumin type. Because legumin-type seed storage proteins vary in size, the 11-12S globulins are collectively referred to as legumins in other species (Balzotti et al., 2008). In our study, proteins belonging to this category were the 11S globulin A or B, and the legumin β-like. The 11S globulins are the major storage proteins in most legumes and in many other dicots (e.g., brassicas, composites, and cucurbits) and in some cereals (oats and rice; Shewry et al., 1995). Also known as chenopodin, the 11S globulin is the major seed storage protein of quinoa. We found two different subunits of chenopodin; A and B subunit. The 11S globulin is a hexamer consisting of six pairs of acidic (34–36 kDa) and basic subunits (22–24 kDa), with each subunit pair connected by a disulfide bond (Brinegar and Goundan, 1993). Recently, the quinoa 11S globulin gene has been suggested to belong to a multigene family (Stevens et al., 2006), as in other species (Shewry et al., 1995). These proteins are related to each other based on their primary structures, with homologies reaching 63% between soybean proA1aB1b and pea prolegumin (Tandang-Silvas et al., 2010).

In the present paper, the 11S seed storage globulin displayed the strongest decrease in salt-treated samples as compared with controls; in fact, in all analyses we found a substantial down-regulation of these proteins and even a complete absence of the corresponding spots in 2DE gels. The decrease in total protein content (Ruiz et al., 2016a) and in percentage content of the albumin/globulin fraction is thus ascribable to the severe reduction of the major seed storage protein, chenopodin. Decreased protein content with increasing salinity can also be attributed to disturbance in nitrogen metabolism or inhibition of nitrate absorption through reduced water uptake and decreased root permeability (Karyotis et al., 2003; Pulvento et al., 2012). The legumin β-like protein displayed an opposite trend insofar as the spot intensity increased in salt-treated samples. Legumin holoproteins are composed of six nearly identical subunits with molecular weights of 50–60 kDa. Each subunit is composed of two differently sized polypeptide chains. The larger more hydrophilic one (30–40 kDa) is named α-chain, whereas the smaller more hydrophobic one (20 kDa) is named β-chain (Müntz, 1996). A relatively lower decrease in energy-rich storage compounds, such as storage proteins (legumin-like and 11S seed storage protein) was found in tolerant wheat genotypes than in less tolerant and sensitive genotypes upon cold treatment (Kosová et al., 2015). In soybean seeds, β-conglycinin and glycinin are the two major storage proteins; sulfur deficiency caused a substantial decrease in the level of glycinins and a concomitant increase in β-conglycinins; the subunit composition of these proteins was also affected (Gayler and Sykes, 1985). The crude acid-soluble globulin seemed not to be affected by growing quinoa under salinity, but since a crude extract and not a purified or highly enriched fraction (as was done for the 11S globulin) was analyzed, we cannot exclude possible changes in protein expression and content. Prolamin-related proteins likewise did not show evident changes in the presence of salt. This is probably due to the low amount of these peptides in gluten-free seeds such as those of quinoa. However, also in this case, we cannot exclude slight changes in this fraction, which represents a very interesting research topic with respect to gluten allergy also in so-called gluten-free crops.

### Amino acid profiles

The amino acid profile of quinoa is one of its major nutritional attributes as it contains all the essential amino acids (Escuredo et al., 2014). The amino acid composition of quinoa seeds of cultivars from the Andean highlands (Bolivia/Argentina) and Argentinian Northwest were shown to vary depending on genotype and area of cultivation (González et al., 2011). Changes in the amino acid profile under drought and salinity, alone or combined, have been described in wild and cultivated barleys (Ahmed et al., 2013), but not in quinoa. In barley, the contents of all amino acids (except Met) were significantly increased relative to controls under drought alone or under combined stress in all genotypes, whereas they decreased or remained unchanged under salinity alone. Present results confirm the strongly genotype-dependent amino acid profile, both under control and salinized conditions, in the three Chilean landraces analyzed. Unexpectedly, the profiles of R49 (*salares* ecotype) and VR (coastal lowlands ecotype) were more similar than those of VR and VI-1, which belong to the same ecotype.

The non-essential amino acids Ala, Pro, and Cys were generally the most abundant PAAs in all three landraces. With the exception of VI-1, Pro was also the most abundant free amino acid; it was one of the few whose content was unaltered or enhanced by salinity in all three landraces, including VI-1. Induction of free amino acids in response to abiotic stress is thought to play a role in plant stress tolerance because of their role as osmolytes. The accumulation of Gly, Pro, Ala, and Val is regarded as a general response to stress. In particular, the accumulation of branched-chain amino acids (BCAAs)—Val, Leu, Ile—is induced by osmotic stress (Joshi et al., 2010). Ile is synthesized from Thr and Met; these three amino acids are synthesized in a highly regulated network depending on physiological and growth requirements. In the present study, free BCAAs (Val, Leu, Ile) were most strongly induced (two- to almost four-fold relative to controls) by salinity in R49, but either not affected or diminished in the other two landraces. It is noteworthy that Thr was also strongly upregulated by salinity in both R49 and VR, but not in VI-1. Unlike Pro, little is known about the function of the stress-induced response involving BCAAs, even though Pro may represent only 10% of the total free amino acids induced by drought (Shen et al., 1989). The increases in BCAAs of drought-stressed tomato leaves were also higher than that of Pro (Joshi et al., 2010). Moreover, it has been proposed that accumulation of free BCAAs may serve as a substrate for the synthesis of stress-induced proteins and that they may act as signaling molecules to regulate gene expression. Further research is needed in this area particularly for stress-tolerant species such as quinoa.

As a general response to abiotic stress, all plants, even halophytes such as quinoa, accumulate amino acids, betaines, sugars, organic acids, and other osmoprotectants (Parida and Das, 2005; Ruiz-Carrasco et al., 2011). Thus, amino acid metabolism, including protease activation, may play an important role in plant tolerance, e.g., under drought stress (Joshi et al., 2010). As observed in the present study, increased contents of at least some amino acids could be the result of protein degradation, although *de novo* synthesis cannot be excluded. For example, Pro decreased in the protein fraction, but increased in the free form in all three landraces of quinoa under salinity. A better understanding of this process will also provide new insight into the negative vs. beneficial effects of abiotic stress on the nutritional qualities of quinoa.

In many cereals, two essential amino acids, Thr and Met, are less abundant than required for the human diet. In R49, free Thr was strongly induced by salinity; similarly, Met was induced by salt in both fractions (free and PAA) only in R49, suggesting that salt stress could improve the nutritional properties of this landrace. By contrast, Thr was very high in VI-1 under non-saline conditions and fell below detection limit in seeds from salt-grown plants. In quinoa, the amount of the essential amino acid Lys is higher than that of cereals (Escuredo et al., 2014). In our study, Lys, representing about 10% of total free amino acids in control seeds, was also differentially affected by salinity in a genotype-dependent manner. Overall, this important amino acid was maintained or induced only in R49 in both fractions.

Based on these results, VI-1 seems to be the most sensitive to salinity in terms of amino acid profiles, and R49 the most tolerant, in accord with its origin from a highly stress-prone area. The differential response between the two coastal-lowlands landraces is in agreement with results for other parameters, namely AA, TPC, and TFC.

### Bioactive molecules and antioxidant activity of quinoa seed protein extracts

Plant-derived antioxidants (phenolic compounds, tocopherols, tocotrienols, ascorbic acid and carotenoids) are essential for counteracting oxidative stress and hence contrasting the insurgence of various diseases. In recent years, the quest for natural antioxidants for dietary as well as cosmetic and pharmaceutical uses is a major objective in plant research. AA is also a matter of great practical interest in food science since oxidation, mainly of lipids and proteins, can lead to the deterioration of quality attributes, such as flavor, aroma, and color. The health benefits of quinoa seeds derive from their nutritional properties (e.g., amino acid composition, minerals, vitamins) but also from their content in bioactive molecules, such as phenolic compounds, and their antioxidant activity. Not surprisingly, therefore, these aspects are increasingly attracting the attention of researchers (Abderrahim et al., 2015; Tang et al., 2015).

We have previously observed that TPC (measured with the FC method) in methanolic extracts of quinoa seed was genotype-dependent and enhanced by salinity in landraces R49 and VR (Ruiz et al., 2016a). In R49, increased seed TPC could be related to better germination capacity on saline media. Improved antioxidant defense may also provide protection from salt stress during seedling establishment. Present results confirm this trend also for PCs obtained from the same seeds. Thus, in all three landraces, seed PCs from plants irrigated with 300 mM NaCl had higher phenolic contents than those grown without NaCl. Spectrophotometric redox assays are among the most common methods for determining total phenolics in plant extracts as they detect the easily oxidized phenolic groups. While being the standard method for the determination of TPC, the FC assay is essentially a means of evaluating the redox potential of a plant extract and is, therefore, subject to interference by many compounds (e.g., sugars, proteins, organic acids; Rodriguez-Amaya, 2010). The Prussian blue method is less subject to interference from non-phenolic compounds and was, therefore, used in parallel in the present work to check for the presence of polyphenols in seed protein extracts. This method provided further proof that polyphenols were present. Although in most cases below detection limit or very low, they were dramatically enhanced in VR under salinity, thus confirming results of the FC assay.

Although protein extraction methods can also extract phenolic compounds, it is important to determine whether they maintain their bioactivity (Salgado et al., 2012). Based on the ABTS radical scavenging test, quinoa seed PC did exhibit AA. Values were also significantly different between landraces (highest in VR) and were slightly increased by salinity in R49 and VR, the two landraces in which TPC and flavonoids (the latter only in VR) also increased the most. These data point to some degree of correlation between phenolic content and AA. If, however, we consider the FC assay also as an indicator of redox potential, then it can be inferred that the antioxidant capacity of salt-treated samples was higher than that of controls in all three landraces.

Phenolic compounds are mainly accumulated in the epidermis to protect photosynthetic tissues from excessive radiation. They also scavenge free radicals and other reactive species insofar as they possess many hydroxyl groups with a high capacity to scavenge ABTS and DPPH (2,2-diphenyl-1-picrylhydrazyl) radicals (Cai et al., 2006). To our knowledge, this is the first time that phenolic content and AA is determined for quinoa seed PCs. In seeds of *A. cruentus*, Escudero et al. (2011) reported TPC values of ca. 74 and 190 mg 100 g^−1^ DW for flour and PC, respectively; values of ca. 39 mg 100 g^−1^ DW in *A. caudatus* and 56 mg 100 g^−1^ DW in *A. paniculatus* have also been reported (Klimczak et al., 2002). Based on the same FC assay, TPC for quinoa seeds (10–70 mg 100 g DW^−1^) were within the range of those reported above for amaranth flour extracts, but lower than those of the PC (prepared essentially as in the present work for quinoa seeds). Total flavonoids in *A. cruentus* (Escudero et al., 2011) were comparable to those of quinoa for seeds from plants grown under non-saline conditions, but were several fold higher in salt-treated quinoa seeds.

A strong correlation between TPC/TFC and AA has been reported in quinoa (Dini et al., 2010; Ismail et al., 2016). The concentration of phenolic compounds in quinoa protein extracts—as determined by the Prussian blue method—was too low to be regarded as the main component involved in AA, except possibly in VR from salt-treated plants. Their presence in proteins extracted by alkalinization followed by acidification may be attributed to a strong interaction between proteins and these secondary metabolites. Seeds, and plant material in general, are also rich in insoluble phenols, simple or highly polymerized (for example, tannins), which can be associated to carbohydrates and proteins (Ozdal et al., 2013). However, there is mounting evidence that proteins themselves can act as free radicals scavengers. The AA of proteins is due to complex interactions between their ability to inactivate ROS, scavenge free radicals, chelate pro-oxidative transition metals, reduce hydroperoxides, enzymatically eliminate specific oxidants, and alter the physical properties of food systems in a way that separates reactive species (Elias et al., 2008). This capacity depends on the amounts of hydrophobic and aromatic amino acids (Petchiammal and Hopper, 2014). The major seed storage proteins of legumes and other plant species have been reported to have radical scavenging potential. In hemp seed, edestin, a storage protein with all the typical features of the 11S globulin storage proteins, was reported to possess antioxidant and antihypertensive activity (Girgih et al., 2014). Present data indicate that PCs from quinoa seeds also have this potential. Moreover, a low but detectable AA was also measured in the 11S (chenopodin) fraction extracted without β-mercaptoethanol.

Establishing a link between salinity tolerance and accumulation of antioxidant molecules is not straightforward. Recently, foliar levels of antioxidant enzymes and molecules in two genotypes of quinoa (cvs Utusaya and Titicaca) with different levels of salinity tolerance were analyzed (Ismail et al., 2016). AA and TPC were slightly increased and rutin concentrations increased by ca. 25-fold in salt-exposed leaves of cv. Titicaca but not Utusaya. These results were interpreted as an indication that the more tolerant genotype Utusaya has a lesser requirement to trigger the accumulation of antioxidant molecules. In accord with this interpretation, foliar levels of polyphenols in control plants of R49 were lower than in VI-1 and VR (Ruiz et al., 2016a). Present results provide a similar picture: VR, the landrace from the less stress-prone area (presumably the least tolerant of the three), exhibited the highest TPC and AA both under control and saline conditions and the highest TFC in the latter situation; by contrast, the tolerant *salares* landrace had the lowest values. Interestingly, the two coastal-lowlands landraces (VI-1 and VR) responded differently to salt treatment; PCs of VR seeds exhibited the stronger salt-induced increase in TPC, TFC, and AA. However, when comparing the *salares* landrace R49 with VR, no major differences could be observed in relative terms (treated vs. control), suggesting that the positive effects of salinity on the amount of bioactive molecules in quinoa PC could be regarded as a general effect and not an indicator of greater or lesser tolerance based on the habitat of origin. This confirms data from another study comparing two genotypes (Regalona and VR) grown under different environmental conditions (arid and cold-temperate), which indicated that nutritional and functional features were enhanced by cultivation in the more stressful arid region (Miranda et al., 2013).

## Conclusions

In conclusion, the data presented here reveal that salinity induced deep changes in the amino acid composition and in protein profiles of the main seed storage proteins of quinoa as well as in the contents of bioactive molecules. The responses were differentially induced in the different landraces, providing evidence that breeding can further ameliorate the nutritional quality of quinoa. The AA of seed protein extracts can be explained by the presence of phenolics, but we cannot exclude that proteins themselves, including the 11S fraction, possess this capacity. The proteomic analysis highlights some promising novel candidates with regard to salt-induced effects on seed quality. The most interesting ones should be further studied in terms of their structural and functional roles in order to enhance our understanding of the salt stress responses in a crop with such unique environmental adaptive characteristics and nutritional value. The strongly genotype-dependent responses to salinity confirm that quinoa landraces are a rich source of genetic variation with respect to stress tolerance and that they are useful for further improving adaptation of this species to diverse environments. Moreover, they confirm that in some cases (e.g., R49) abiotic stress may improve the nutritional properties of quinoa seeds.

## Author contributions

KR designed the experiment and produced the plant material; IA, KR, CL, and LP performed the experiments, analyzed the data and prepared the figures; SB, SD, GC, and LB planned the research and interpreted the data; IA, SB, and LP wrote the article with contributions from all other authors.

### Conflict of interest statement

The authors declare that the research was conducted in the absence of any commercial or financial relationships that could be construed as a potential conflict of interest.

## References

[B1] Abderrahim F. Huanatico E. Segura R. Arribas S. Gonzalez M. C. Condezo-Hoyos L. (2015). Physical features, phenolic compounds, betalains and total antioxidant capacity of coloured quinoa seeds (*Chenopodium quinoa* Willd.) from Peruvian Altiplano. Food Chem. 183, 83–90. 10.1016/j.foodchem.2015.03.029 25863614

[B2] Abugoch L. E. Romero N. Tapia C. A. Silva J. Rivera M. (2008). Study of some physicochemical and functional properties of quinoa (*Chenopodium quinoa* Willd.) protein isolates. J. Agric. Food Chem. 56, 4745–4750. 10.1021/jf703689u 18489119

[B3] Adolf V. I. Jacobsen S.-E. Shabala S. (2013). Salt tolerance mechanisms in quinoa (*Chenopodium quinoa* Willd.). Environ. Exp. Bot. 92, 43–54. 10.1016/j.envexpbot.2012.07.004

[B4] Adolf V. I. Shabala S. Andersen M. N. Razzaghi F. Jacobsen S.-E. (2012). Varietal differences of quinoa's tolerance to saline conditions. Plant Soil 357, 117–129. 10.1007/s11104-012-1133-7

[B5] Ahmad F. Slinkard A. E. (1992). Genetic relationships in the genus *Cicer* L. as revealed by polyacrylamide gel electrophoresis of seed storage proteins. Theor. Appl. Genet. 84, 688–692. 10.1007/bf00224169 24201358

[B6] Ahmed I. M. Cao F. Han Y. Nadira U. A. Zhang G. Wu F. (2013). Differential changes in grain ultrastructure, amylase, protein and amino acid profiles between Tibetan wild and cultivated barleys under drought and salinity alone and combined stress. Food Chem. 141, 2743–2750. 10.1016/j.foodchem.2013.05.101 23871019

[B7] Arnao M. B. Cano A. Alcolea J. F. Acosta M. (2001). Estimation of free radical-quenching activity of leaf pigment extracts. Phytochem. Anal. 12, 138–143. 10.1002/pca.571 11705243

[B8] Balzotti M. R. B. Thornton J. N. Maughan P. J. McClellan D. A. Stevens M. R. Jellen E. N. (2008). Expression and evolutionary relationships of the *Chenopodium quinoa* 11S seed storage protein gene. Int. J. Plant Sci. 169, 281–291. 10.1086/523874

[B9] Bergamo P. Maurano F. Mazzarella G. Iaquinto G. Vocca I. Rivelli A. R.. (2011). Immunological evaluation of the alcohol-soluble protein fraction from gluten-free grains in relation to celiac disease. Mol. Nutr. Food Res. 55, 1266–1270. 10.1002/mnfr.201100132 21710563

[B10] Bhargava A. Rana T. S. Shukla S. Ohri D. (2005). Seed protein electrophoresis of some cultivated and wild species of *Chenopodium*. Biol. Plant. 49, 505–511. 10.1007/s10535-005-0042-5

[B11] Brinegar C. Goundan S. (1993). Isolation and characterization of chenopodin, the 11S seed storage protein of quinoa (*Chenopodium quinoa*). J. Agric. Food Chem. 41, 182–185. 10.1021/jf00026a006

[B12] Brinegar C. Sine B. Nwokocha L. (1996). High-cysteine 2S seed storage proteins from quinoa (*Chenopodium quinoa*). J. Agric. Food Chem. 44, 1621–1623. 10.1021/jf950830+

[B13] Burrieza H. P. Koyro H.-W. Martínez-Tosar L. Kobayashi K. Maldonado S. (2012). High salinity induces dehydrin accumulation in *Chenopodium quinoa* Willd. cv. Hualhuas embryos. Plant Soil 354, 69–79. 10.1007/s11104-011-1045-y

[B14] Cai Y. Z. Mei S. Jie X. Luo Q. Corke H. (2006). Structure-radical scavenging activity relationships of phenolic compounds from traditional Chinese medicinal plants. Life Sci. 78, 2872–2888. 10.1016/j.lfs.2005.11.004 16325868

[B15] Cao W. H. Liu J. He X. J. Mu R. L. Zhou H. L. Chen S. Y.. (2007). Modulation of ethylene responses affects plant salt-stress responses. Plant Physiol. 143, 707–719. 10.1104/pp.106.094292 17189334 PMC1803741

[B16] Caruso G. Cavaliere C. Guarino C. Gubbiotti R. Foglia P. Lagana A. (2008). Identification of changes in *Triticum durum* L. leaf proteome in response to salt stress by two-dimensional electrophoresis and MALDI-TOF mass spectrometry. Anal. Bioanal. Chem. 391, 381–390. 10.1007/s00216-008-2008-x 18365183

[B17] Castel V. Andrich O. Netto F. M. Santiago L. G. Carrara C. R. (2014). Total phenolic content and antioxidant activity of different streams resulting from pilot-plant processes to obtain *Amaranthus mantegazzianus* protein concentrates. J. Food Eng. 122, 62–67. 10.1016/j.jfoodeng.2013.08.032

[B18] Cordero-De-Los-Santos J. Osuna-Castro A. Borodanenko A. Paredes-López O. (2005). Physicochemical and functional characterization of Amaranth (*Amaranthus hypochondriacus*) protein isolates obtained by isoelectric precipitation and micellisation. Food Sci. Technol. Int. 11, 269–280. 10.1177/1082013205056491

[B19] Dini I. Tenore G. C. Dini A. (2010). Antioxidant compound contents and antioxidant activity before and after cooking in sweet and bitter *Chenopodium quinoa* seeds. LWT Food Sci. Technol. 43, 447–451. 10.1016/j.lwt.2009.09.010

[B20] Džunková M. Janovská D. Čepková P. H. Prohasková A. Kolář M. (2011). Glutelin protein fraction as a tool for clear identification of amaranth accessions. J. Cereal Sci. 53, 198–205. 10.1016/j.jcs.2010.12.003

[B21] Elias R. J. Kellerby S. S. Decker E. A. (2008). Antioxidant activity of proteins and peptides. Crit. Rev. Food Sci. Nutr. 48, 430–441. 10.1080/10408390701425615 18464032

[B22] El-Iklil Y. Karrou M. Benichou M. (2000). Salt stress effect on epinasty in relation to ethylene production and water relations in tomato. Agronomie 20, 399–406. 10.1051/agro:2000136

[B23] Escudero N. L. Albarracín G. J. Lucero López R. V. Giménez M. S. (2011). Antioxidant activity and phenolic content of flour and protein concentrate of *Amaranthus cruentus* seeds. J. Food Biochem. 35, 1327–1341. 10.1111/j.1745-4514.2010.00454.x

[B24] Escudero N. L. Arellano M. L. Luco J. Giménez M. S. Mucciarelli S. (2004). Comparison of the chemical composition and nutritional value of *Amaranthus cruentus* flour and protein concentrate. Plant Foods Hum. Nutr. 59, 15–21. 10.1007/s11130-004-0033-3 15675147

[B25] Escuredo O. González Martín M. I. Moncada G. W. Fischer S. Hernández Hierro J. M. (2014). Amino acid profile of the quinoa (*Chenopodium quinoa* Willd.) using near infrared spectroscopy and chemometric techniques. J. Cereal Sci. 60, 67–74. 10.1016/j.jcs.2014.01.016

[B26] Fuentes F. F. Martínez E. A. Hinrichsen P. V. Jellen E. N. Maughan P. J. (2009). Assessment of genetic diversity patterns in Chilean quinoa (*Chenopodium quinoa* Willd.) germplasm using multiplex fluorescent microsatellite markers. Conserv. Genet. 10, 369–377. 10.1007/s10592-008-9604-3

[B27] Gayler K. R. Sykes G. E. (1985). Effects of nutritional stress on the storage proteins of soybeans. Plant Physiol. 78, 582–585. 10.1104/pp.78.3.582 16664286 PMC1064779

[B28] Ghafoor A. Ahmad Z. Qureshi A. S. Bashir M. (2002). Genetic relationship in *Vigna mungo* (L.) Hepper and *V. radiata (L.)* R. Wilczek based on morphological traits and SDS-PAGE. Euphytica 123, 367–378. 10.1023/A:1015092502466

[B29] Girgih A. T. He R. Malomo S. Offengenden M. Wu J. Aluko R. E. (2014). Structural and functional characterization of hemp seed (*Cannabis sativa* L.) protein derived antioxidant and antihypertensive peptides. J. Funct. Foods 6, 384–394. 10.1016/j.jff.2013.11.005

[B30] Gómez-Caravaca A. Iafelice G. Lavini A. Pulvento C. Caboni M. Marconi E. (2012). Phenolic compounds and saponins in quinoa samples (*Chenopodium quinoa* Willd.) grown under different saline and nonsaline irrigation regimes. J. Agric. Food Chem. 60, 4620–4627. 10.1021/jf3002125 22512450

[B31] Gómez-Pando L. R. Álvarez-Castro R. De La Barra E. (2010). Effect of salt stress on Peruvian germplasm of *Chenopodium quinoa* Willd.: a promising crop. J. Agron. Crop Sci. 196, 391–396. 10.1111/j.1439-037X.2010.00429.x

[B32] González J. A. Konishi Y. Bruno M. Valoy M. Prado F. E. (2011). Interrelationships among seed yield, total protein and amino acid composition of ten quinoa (*Chenopodium quinoa*) cultivars from two different agroecological regions. J. Sci. Food Agr. 92, 1222–1229. 10.1002/jsfa.4686 22002725

[B33] Hagerman A. E. Butler L. G. (1994). Assay of condensed tannins or flavonoid oligomers and related flavonoids in plants. Methods Enzymol. 234, 429–437. 10.1016/0076-6879(94)34113-3 7808315

[B34] Hariadi Y. Marandon K. Tian Y. Jacobsen S.-E. Shabala S. (2011). Ionic and osmotic relations in quinoa (*Chenopodium quinoa* Willd.) plants grown at various salinity levels. J. Exp. Bot. 62, 185–193. 10.1093/jxb/erq257 20732880 PMC2993909

[B35] Hellman U. Wernstedt C. Gonez J. Heldin C. H. (1995). Improvement of an “In-Gel” digestion procedure for the micropreparation of internal protein fragments for amino acid sequencing. Anal. Biochem. 224, 451–455. 10.1006/abio.1995.1070 7710111

[B36] Hirose Y. Fujita T. Ishii T. Ueno N. (2010). Antioxidative properties and flavonoid composition of *Chenopodium quinoa* seeds cultivated in Japan. Food Chem. 119, 1300–1306. 10.1016/j.foodchem.2009.09.008

[B37] Ismail H. Dragiši Maksimović H. Maksimović V. Shabala L. Živanović B. D. Tian Y. (2016). Rutin, a flavonoid with antioxidant activity, improves plant salinity tolerance by regulating K^+^ retention and Na^+^ exclusion from leaf mesophyll in quinoa and broad beans. Funct. Plant Biol. 43, 75–86. 10.1071/FP15312 32480443

[B38] Jacobsen S. E. Liu F. Jensen C. R. (2009). Does root-sourced ABA play a role for regulation of stomata under drought in quinoa (*Chenopodium quinoa* Willd.). Sci. Hortic. 122, 281–287. 10.1016/j.scienta.2009.05.019

[B39] Jacobsen S.-E. Mujica A. Jensen C. R. (2003). The resistance of quinoa (*Chenopodium quinoa* Willd.) to adverse abiotic factors. Food Rev. Int. 19, 99–109. 10.1081/FRI-120018872

[B40] Jha S. S. Ohri D. (1996). Phylogenetic relationships of *Cajanus cajan* (L.) Millsp. *(pigeon pea)* and its wild relatives based on seed protein profiles. Genet. Res. Crop Evol. 43, 275–281. 10.1007/BF00123279

[B41] Joshi V. Joung J.-G. Fei Z. Jander G. (2010). Interdependence of threonine, methionine and isoleucine metabolism in plants: accumulation and transcriptional regulation under abiotic stress. Amino Acids 39, 933–947. 10.1007/s00726-010-0505-7 20186554

[B42] Ju Z. Y. Hettiarachchy N. S. Rath N. (2001). Extraction, denaturation and hydrophobic properties of rice flour proteins. J. Food Sci. 66, 229–232. 10.1111/j.1365-2621.2001.tb11322.x

[B43] Karyotis T. Iliadis C. Noulas C. Mitsibonas T. (2003). Preliminary research on seed production and nutrient content for certain quinoa varieties in a saline-sodic soil. J. Agron. Crop Sci. 189, 402–408. 10.1046/j.0931-2250.2003.00063.x

[B44] Khan A. A. Akbar M. Seshu D. V. (1987). Ethylene as an indicator of salt tolerance in rice. Crop Sci. 27, 1242–1248. 10.2135/cropsci1987.0011183X002700060031x

[B45] Klimczak I. Małecka M. Pachołek B. (2002). Antioxidant activity of ethanolic extracts of amaranth seeds. Nahrung/Food 46, 184–186. 10.1002/1521-3803(20020501)46:3<184::AID-FOOD184>3.0.CO;2-H 12108218

[B46] Kosová K. Vítámvás P. Urban M. O. Klíma M. Roy A. Prášil I. T. (2015). Biological networks underlying abiotic stress tolerance in temperate crops-a proteomic perspective. Int. J. Mol. Sci. 16, 20913–20942. 10.3390/ijms160920913 26340626 PMC4613235

[B47] Koyro H.-W. Eisa S. S. (2008). Effect of salinity on composition, viability and germination of seeds of *Chenopodium quinoa* Willd. Plant Soil 302, 79–90. 10.1007/s11104-007-9457-4

[B48] Koyro H.-W. Zörb C. Debez A. Huchzermeyer B. (2013). The effect of hyper-osmotic salinity on protein pattern and enzyme activities of halophytes. Funct. Plant Biol. 40, 787–804. 10.1071/fp12387 32481151

[B49] Laemmli U. K. (1970). Cleavage of structural proteins during assembly of head of bacteriophage-T4. Nature 227, 680–685. 10.1038/227680a0 5432063

[B50] Liu M. Li X. Q. Weber C. Lee C. Y. Brown J. Liu R. H. (2002). Antioxidant and antiproliferative activities of raspberries. J. Agric. Food Chem. 50, 2926–2930. 10.1021/jf0111209 11982421

[B51] Miranda M. Delatorre-Herrera J. Vega-Gálvez A. Jorquera E. Quispe-Fuentes I. Martínez E. A. (2014). Antimicrobial potential and phytochemical content of six diverse sources of quinoa seeds (*Chenopodium quinoa* Willd.). Agric. Sci. 5, 1015–1024. 10.4236/as.2014.511110

[B52] Miranda M. Vega-Gálvez A. Martínez E. A. López J. Marín R. Aranda M. (2013). Influence of contrasting environments on seed composition of two quinoa genotypes: nutritional and functional properties. Chil. J. Agric. Res. 73, 108–116. 10.4067/S0718-58392013000200004

[B53] Müntz K. (1996). Proteases and proteolytic cleavage of storage proteins in developing and germinating dicotyledonous seeds. J. Exp. Bot. 47, 605–622. 10.1093/jxb/47.5.605

[B54] Nath P. Ohri D. Jha S. S. Pal M. (1997). Seed protein electrophoresis of wild and cultivated species of *Celosia* (Amaranthaceae). Genet. Res. Crop Evol. 44, 241–245. 10.1023/A:1008670407477

[B55] Ndimba B. K. Chivasa S. Simon W. J. Slabas A. R. (2005). Identification of Arabidopsis salt and osmotic stress responsive proteins using two-dimensional difference gel electrophoresis and mass spectrometry. Proteomics 5, 4185–4196. 10.1002/pmic.200401282 16254930

[B56] Osborne T. B. (1924). The Vegetable Proteins. London: Longmans, Green.

[B57] Ozdal T. C. Apanoglu E. Altay F. (2013). A review on protein–phenolic interactions and associated changes. Food Res. Int. 51, 954–970. 10.1016/j.foodres.2013.02.009

[B58] Parida A. K. Das A. B. (2005). Salt tolerance and salinity effects on plants: a review. Ecotoxicol. Environ. Saf. 60, 324–349. 10.1016/j.ecoenv.2004.06.010 15590011

[B59] Paśko P. Bartoń H. Zagrodzki P. Gorinstein S. Fołta M. Zachwieja Z. (2009). Anthocyanins, total polyphenols and antioxidant activity in amaranth and quinoa seeds and sprouts during their growth. Food Chem. 115, 994–998. 10.1016/j.foodchem.2009.01.037

[B60] Petchiammal C. Hopper W. (2014). Antioxidant activity of proteins from fifteen varieties of legume seeds commonly consumed in India. Int. J. Pharm. Sci. 6, 476–479.

[B61] Peterson A. Murphy K. (2015). Tolerance of lowland quinoa cultivars to sodium chloride and sodium sulfate salinity. Crop Sci. 55, 331–338. 10.2135/cropsci2014.04.0271

[B62] Prego I. Maldonado S. Otegui M. (1998). Seed structure and localization of reserves in C*henopodium quinoa*. Ann. Bot. 82, 481–488. 10.1006/anbo.1998.0704

[B63] Pulvento C. Riccardi M. Lavini A. Iafelice G. Marconi E. D'Andria R. (2012). Yield and quality characteristics of *Chenopodium quinoa* Willd. grown in open field under different saline and not saline irrigation. J. Agron. Crop Sci. 198, 254–263. 10.1111/j.1439-037X.2012.00509.x

[B64] Raman S. B. Rathinasabapathi B. (2004). Pantothenate synthesis in plants. Plant Sci. 167, 961–968. 10.1016/j.plantsci.2004.06.019

[B65] Repo-Carrasco-Valencia R. Hellström J. K. Pihlava J.-M. Mattila P. H. (2010). Flavonoids and other phenolic compounds in Andean indigenous grains: Quinoa (*Chenopodium quinoa*), kañiwa (*Chenopodium pallidicaule*) and kiwicha (*Amaranthus caudatus*). Food Chem. 120, 128–133. 10.1016/j.foodchem.2009.09.087

[B66] Rodriguez-Amaya D. B. (2010). Quantitative analysis, *in vitro* assessment of bioavailability and antioxidant activity of food carotenoids - A review. J. Food Comp. Anal. 23, 726–740. 10.1016/j.jfca.2010.03.008

[B67] Rodriguez-Rosales M. P. Jiang X. Galvez F. J. Aranda M. N. Cubero B. Venema K. (2008). Overexpression of the tomato K^+^/H^+^ antiporter *LeNHX2* confers salt tolerance by improving potassium compartmentalization. New Phytol. 179, 366–377. 10.1111/j.1469-8137.2008.02461.x 19086176

[B68] Rubio S. Whitehead L. Larson T. R. Graham I. A. Rodriguez P. L. (2008). The coenzyme A biosynthetic enzyme phosphopantetheine adenylyltransferase plays a crucial role in plant growth, salt/osmotic stress resistance, and seed lipid storage. Plant Physiol. 148, 546–556. 10.1104/pp.108.124057 18621975 PMC2528120

[B69] Ruiz K. B. Aloisi I. Del Duca S. Canelo V. Torrigiani P. Silva H.. (2016a). *Salares* versus coastal ecotypes of quinoa: salinity responses in Chilean landraces from contrasting habitats. Plant Physiol. Biochem. 101, 1–13. 10.1016/j.plaphy.2016.01.010 26841266

[B70] Ruiz K. B. Biondi S. Martínez E. A. Orsini F. Antognoni F. Jacobsen S.-E. (2016b). Quinoa - a model crop for understanding salt tolerance mechanisms in halophytes. Plant Biosyst. 150, 357–371. 10.1080/11263504.2015.1027317

[B71] Ruiz-Carrasco K. B. Antognoni F. Coulibaly A. K. Lizardi S. Covarrubias A. Martínez E. A.. (2011). Variation in salinity tolerance of four lowland genotypes of quinoa (*Chenopodium quinoa* Willd.) as assessed by growth, physiological traits, and sodium transporter gene expression. Plant Physiol. Biochem. 49, 1333–1341. 10.1016/j.plaphy.2011.08.005 22000057

[B72] Salgado P. R. López-Caballero M. E. Gómez-Guillén M. C. Mauri A. N. Montero M. P. (2012). Exploration of the antioxidant and antimicrobial capacity of two sunflower protein concentrate films with naturally present phenolic compounds. Food Hydrocoll. 29, 374–381. 10.1016/j.foodhyd.2012.03.006

[B73] Sanan-Mishra N. Pham X. H. Sopory S. K. Tuteja N. (2005). Pea DNA helicase 45 overexpression in tobacco confers high salinity tolerance without affecting yield. Proc. Natl. Acad. Sci. U.S.A. 102, 509–514. 10.1073/pnas.0406485102 15630095 PMC544286

[B74] Sano N. Rajjou L. North H. M. Debeaujon I. Marion-Poll A. Seo M. (2015). Staying alive: molecular aspects of seed longevity. Plant Cell Physiol. 57, 660–674. 10.1093/pcp/pcv186 26637538

[B75] Schwender J. Goffman F. Ohlrogge J. B. Shachar-Hill Y. (2004). Rubisco without the Calvin cycle improves the carbon efficiency of developing green seeds. Nature 432, 779–782. 10.1038/nature03145 15592419

[B76] Shen L. Foster J. G. Orcutt D. M. (1989). Composition and distribution of free amino-acids in flatpea (*Lathyrus sylvestris* L.) as influenced by water deficit and plant-age. J. Exp. Bot. 40, 71–79. 10.1093/jxb/40.1.71

[B77] Shevchenko A. Wilm M. Vorm O. Mann M. (1996). Mass spectrometric sequencing of proteins silver-stained polyacrylamide gels. Anal. Chem. 68, 850–858. 10.1021/ac950914h 8779443

[B78] Shewry P. R. Napier J. A. Tatham A. S. (1995). Seed storage proteins: structures and biosynthesis. Plant Cell 7, 945–956. 10.1105/tpc.7.7.945 7640527 PMC160892

[B79] Silvanini A. Dall'Asta C. Morrone L. Cirlini M. Beghè D. Fabbri A. (2014). Altitude effects on fruit morphology and flour composition of two chestnut cultivars. Sci. Hortic. 176, 311–318. 10.1016/j.scienta.2014.07.008

[B80] Singleton V. L. Rossi J. A. J. (1965). Colorimetry of total phenolics with phosphomolybdic-phosphotungstic acid reagent. Am. J. Enol. Vitic. 16, 144–158.

[B81] Soskić V. Gorlach M. Poznanović S. Boehmer F. D. Godovac-Zimmermann J. (1999). Functional proteomics analysis of signal transduction pathways of the platelet-derived growth factor beta receptor. Biochemistry 38, 1757–1764. 10.1021/bi982093r 10026255

[B82] Stevens M. R. Coleman C. E. Parkinson S. E. Maughan P. J. Zhang H. B. Balzotti M. R.. (2006). Construction of a quinoa (*Chenopodium quinoa* Willd.) BAC library and its use in identifying genes encoding seed storage proteins. Theor. Appl. Genet. 112, 1593–1600. 10.1007/s00122-006-0266-6 16586115

[B83] Tandang-Silvas M. R. Fukuda T. Fukuda C. Prak K. Cabanos C. Kimura A.. (2010). Conservation and divergence on plant seed 11S globulins based on crystal structures. Biochim. Biophys. Acta 1804, 1432–1442. 10.1016/j.bbapap.2010.02.016 20215054

[B84] Tang Y. Li X. Zhang B. Chen P. X. Liu R. Tsao R. (2015). Characterisation of phenolics, betanins and antioxidant activities in seeds of three *Chenopodium quinoa* Willd. genotypes. Food Chem. 166, 380–388. 10.1016/j.foodchem.2014.06.018 25053071

[B85] Thanh V. H. Okubo K. Shibasaki K. (1975). Isolation and characterization of the multiple 7S globulins of soybean proteins. Plant Physiol. Biochem. 56, 19–22. 10.1104/pp.56.1.19 16659250 PMC541290

[B86] Tilton G. B. Wedemeyer W. J. Ohlrogge J. (2006). Plant coenzyme A biosynthesis: characterization of two pantothenate kinases from Arabidopsis. Plant Mol. Biol. 61, 629–642. 10.1007/s11103-006-0037-4 16897480

[B87] Vashisht A. A. Pradhan A. Tuteja R. Tuteja N. (2005). Cold- and salinity stress-induced bipolar pea DNA helicase 47 is involved in protein synthesis and stimulated by phosphorylation with protein kinase C. Plant J. 44, 76–87. 10.1111/j.1365-313X.2005.02511.x 16167897

[B88] Vega-Gálvez A. Miranda M. Vergara J. Uribe E. Puente L. Martínez E. A. (2010). Nutrition facts and functional potential of quinoa (*Chenopodium quinoa* Willd.), an ancient Andean grain: a review. J. Sci. Food Agric. 90, 2541–2547. 10.1002/jsfa.4158 20814881

[B89] Vidueiros S. M. Curti R. N. Dyner L. M. Binaghi M. J. Peterson G. Bertero H. D. (2015). Diversity and interrelationships in nutritional traits in cultivated quinoa (*Chenopodium quinoa* Willd.) from Northwest Argentina. J. Cereal Sci. 62, 87–93. 10.1016/j.jcs.2015.01.001

[B90] Wang W. Vinocur B. Shoseyov O. Altman A. (2004). Role of plant heat-shock proteins and molecular chaperones in the abiotic stress response. Trends Plant Sci. 9, 244–252. 10.1016/j.tplants.2004.03.006 15130550

[B91] Zevallos V. F. Ellis H. J. Suligoj T. Herencia L. I. Ciclitira P. J. (2012). Variable activation of immune response by quinoa (*Chenopodium quinoa* Willd.) prolamins in celiac disease. Am. J. Clin. Nutr. 96, 337–344. 10.3945/ajcn.111.030684 22760575

[B92] Zhang X. Liu S. Takano T. (2008). Overexpression of a mitochondrial ATP synthase small subunit gene (*AtMtATP6*) confers tolerance to several abiotic stresses in *Saccharomyces cerevisiae* and *Arabidopsis thaliana*. Biotechnol. Lett. 30, 1289–1294. 10.1007/s10529-008-9685-6 18338219

[B93] Zhu J. K. (2003). Regulation of ion homeostasis under salt stress. Curr. Opin. Plant Biol. 6, 441–445. 10.1016/S1369-5266(03)00085-2 12972044

